# Three Hcp homologs with divergent extended loop regions exhibit different functions in avian pathogenic *Escherichia coli*

**DOI:** 10.1038/s41426-018-0042-0

**Published:** 2018-03-29

**Authors:** Jiale Ma, Min Sun, Zihao Pan, Wenchao Song, Chengping Lu, Huochun Yao

**Affiliations:** 1 College of Veterinary Medicine, Nanjing Agricultural University, Nanjing, 210095 China; 2 Key Lab of Animal Bacteriology, Ministry of Agriculture, Nanjing, 210095 China

## Abstract

Type VI secretion systems (T6SSs) contribute to the pathogenicity of avian pathogenic *Escherichia coli* (APEC), one of the leading causative agents of sepsis and meningitis in poultry. The Hcp protein is a core component of the T6SS tail tube and acts as an exported receptor and a chaperone of effectors. In this study, four distinct Hcp types (Ia, Ib, IIa, and IIb) were designated in Gram-negative bacteria, three of which were widely distributed in APEC. We detected divergence in transcription levels among three *hcp* clusters in 50% duck serum and demonstrated that *hcp1* was upregulated by relieving Fur repression. Further analyses revealed that the host serum could activate the *hcp2B* operon by H-NS derepression to transcribe the downstream *xmtU/xmtV* pair for inter-bacterial antagonism. Notably, in a structural analysis based on the genetic classification, Hcp proteins exhibited significant differences in the extended loop regions, suggesting that these regions were related to their functional properties. Indeed, the variant region Vs2 (Loop L2, 3) in Hcp1 and Hcp2B was essential for the delivery of antibacterial effectors and the inhibition of macrophage phagocytosis. Further analyses using a duck model indicated that these Hcps play different roles in the pathogenic processes of APEC and immunoprotection. These results indicated that the functional differentiation of Hcp homologs was driven by differences in transcriptional regulation, extended loop regions, and effector delivery.

## Introduction

Veterinary and medical *Escherichia coli* (*E. coli*) are currently categorized into intestinal pathogenic and extraintestinal pathogenic (ExPEC) isolates^1^. Avian pathogenic *E. coli* (APEC) is an important ExPEC subgroup; these strains are closely related to human ExPEC strains^2, 3^. In fact, mixed infections with APEC, neonatal meningitis *E. coli* (NMEC), and other ExPEC in animals may promote the gradual evolution of virulence in ExPEC^4, 5^.

The type VI secretion system (T6SS) has been determined to be a common virulence factor that is shared between APEC and NMEC; it contributes to multiple processes, ranging from inter-bacterial killing to pathogenesis^6, 7^. However, the molecular mechanisms by which T6SS contributes to *E. coli* pathogenicity are still unclear. Hemolysin coregulated protein (Hcp) is a central component of the T6SS nanomachine and plays critical roles in both the assembly of the T6SS apparatus and the export of its effectors^8, 9^. In fact, most ExPEC strains encode three dissimilar Hcps belonging to distinct T6SS1 and T6SS2 complexes^10^. These Hcps share similar protein sizes and structures but display significant distance in genetics and functions^6, 7, 11^. It is thought that slight modifications in Hcp are required for its biological properties. Indeed, a mutation of two residues within the L2, 3 loop abolishes the stacking of Hcp hexamers to form tubular structures in *Burkholderia pseudomallei* and impairs effector secretion^12^. Further studies in this field will help to clarify the intrinsic mechanisms underlying the functional differentiation of T6SSs.

An increasing number of T6SS effectors involved in inter-bacterial competition have been identified, and numerous effectors (including Hcp, VgrG, and Tle) have been shown to function in interactions with eukaryotic host cells^13–17^. Hcp induces cytotoxicity in *Dictyostelium amoebae* and J774 murine macrophages during *Vibrio cholerae* infection^18^ and facilitates efficient tumorigenesis in *Agrobacterium tumefaciens* infection^19^. Hcp1 is secreted *in vivo* during *Burkholderia mallei* infection in humans and horses and has been detected in the sera of patients with cystic fibrosis^20^. Thus, Hcp-like proteins have specific pathogenic roles in many bacterial diseases, but their roles in APEC infection are unclear.

In this study, we examined the details of three distinct Hcp proteins in two functional T6SS clusters of the APEC strain TW-XM. Notably, these three *hcp* clusters showed different regulatory mechanisms at the transcriptional level and had different effects on inter-bacterial competition, biofilm formation in serum, and immunoprotection against lethal challenges with APEC. Furthermore, two variant sequence regions, Vs1 (in Loop L1, 2) and Vs2 (in Loop L2, 3), were identified in different Hcp types, and Vs2 was responsible for the functions of Hcp1 and Hcp2B in the transportation of antibacterial effectors and interactions with host cells.

## Results

### Genetic classification of Hcp proteins

The *hcp* gene is widely distributed in the genomes of Gram-negative bacteria, while its classification is still unclear. In fact, Hcp proteins share similar structures and functions in the T6SS machineries from different bacterial species^12, 21^ but display significant differences in genetics, particularly in the extended loop regions^12^. In this study, a phylogenetic tree of Hcp proteins from diverse bacterial species was constructed, and four deep branches were observed (designated type Ia, Ib, IIa, and IIb, Supplementary Fig. S1). Notably, Hcp1, Hcp2A, and Hcp2B of APEC isolates belonged to type Ib, IIa, and IIb Hcp homologs, respectively (Supplementary Fig. S1).

### Secretion identification of three Hcps in APEC *in vivo*

**Fig. 1:**
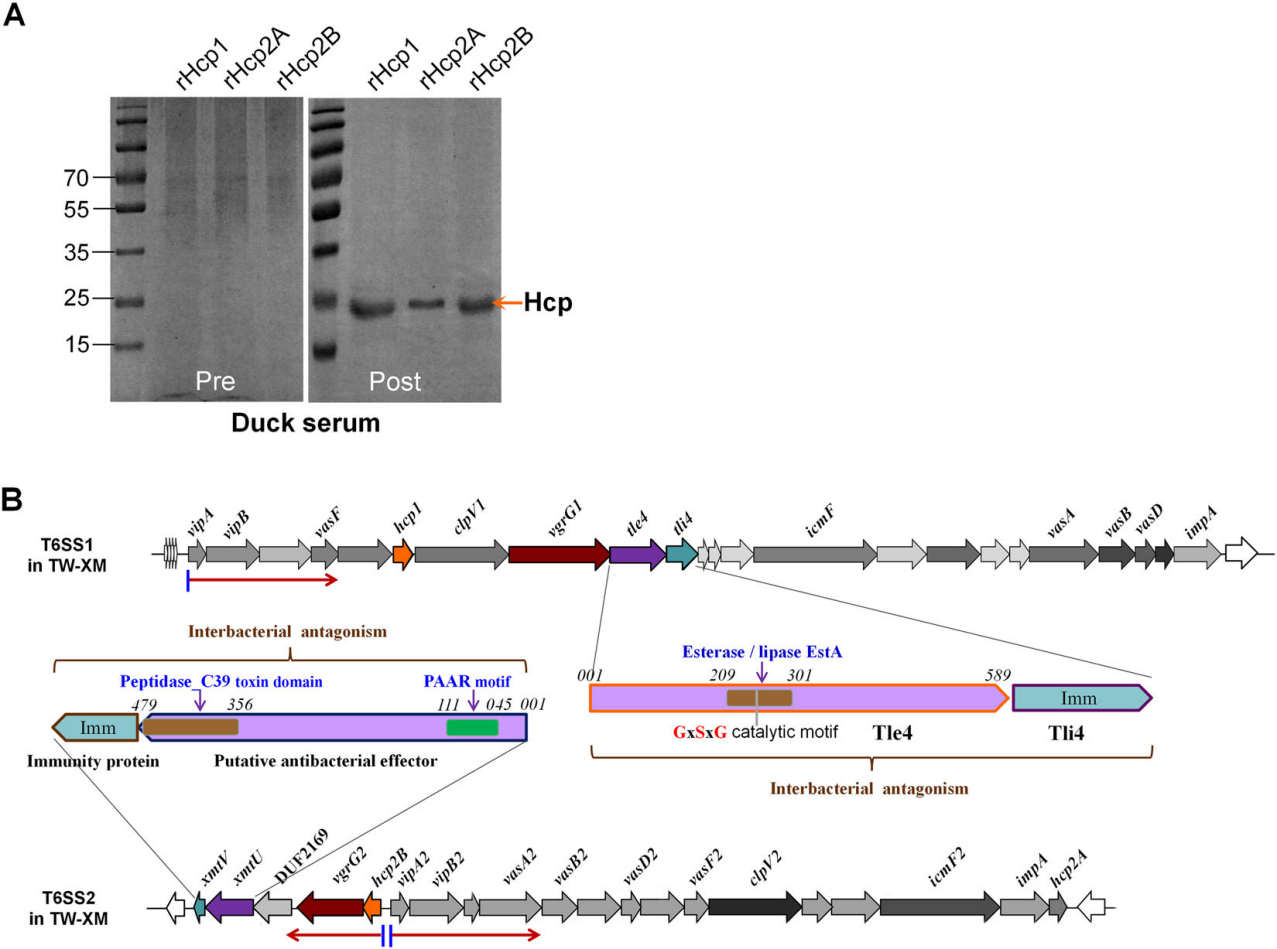
Immunoblotting analyses of Hcp family proteins and genetic organization of T6SS loci in APEC. **a** Immunoblotting with anti-Hcps antibodies using purified rHcps in convalescent serum. The ducks were infected with 2 × LD_50_ APEC TW-XM, and sera from the surviving ducks were collected after 2 weeks. Pre-immune serum was used as a negative control. **b** Genetic organization of the T6SS1 and T6SS2 loci in APEC. The transcription directions are indicated by a red arrow. The domain architectures of the antibacterial effectors XmtU and Tle4 are denoted by different colors and annotations

It is not clear whether all three Hcps from APEC are functional *in vivo*. Some previous studies have confirmed that Hcp2B is not secreted into cell supernatants but only exists in bacterial lysates at low levels when APEC are cultured in LB-rich medium^7^. Three recombinant Hcp proteins (rHcp) were purified (Supplementary Fig. S2), and their immune sera were analyzed, indicating no distinct cross-reactivity among different Hcp types by indirect enzyme-linked immunosorbent assay (ELISA; Supplementary Fig. S3), which suggested a specific host antibody response to immunization with different Hcps. Further study showed that specific anti-Hcp1, anti-Hcp2A, and anti-Hcp2B antibodies could be detected in the sera of surviving ducklings challenged with a sub-lethal dose of APEC (Fig. 1b). These results suggested that all three Hcps were assembled in the T6SS complexes and secreted into the extracellular matrix to activate humoral immunity during duck infection.

### Hcp1 production was activated by relieving Fur repression in duck serum

**Fig. 2:**
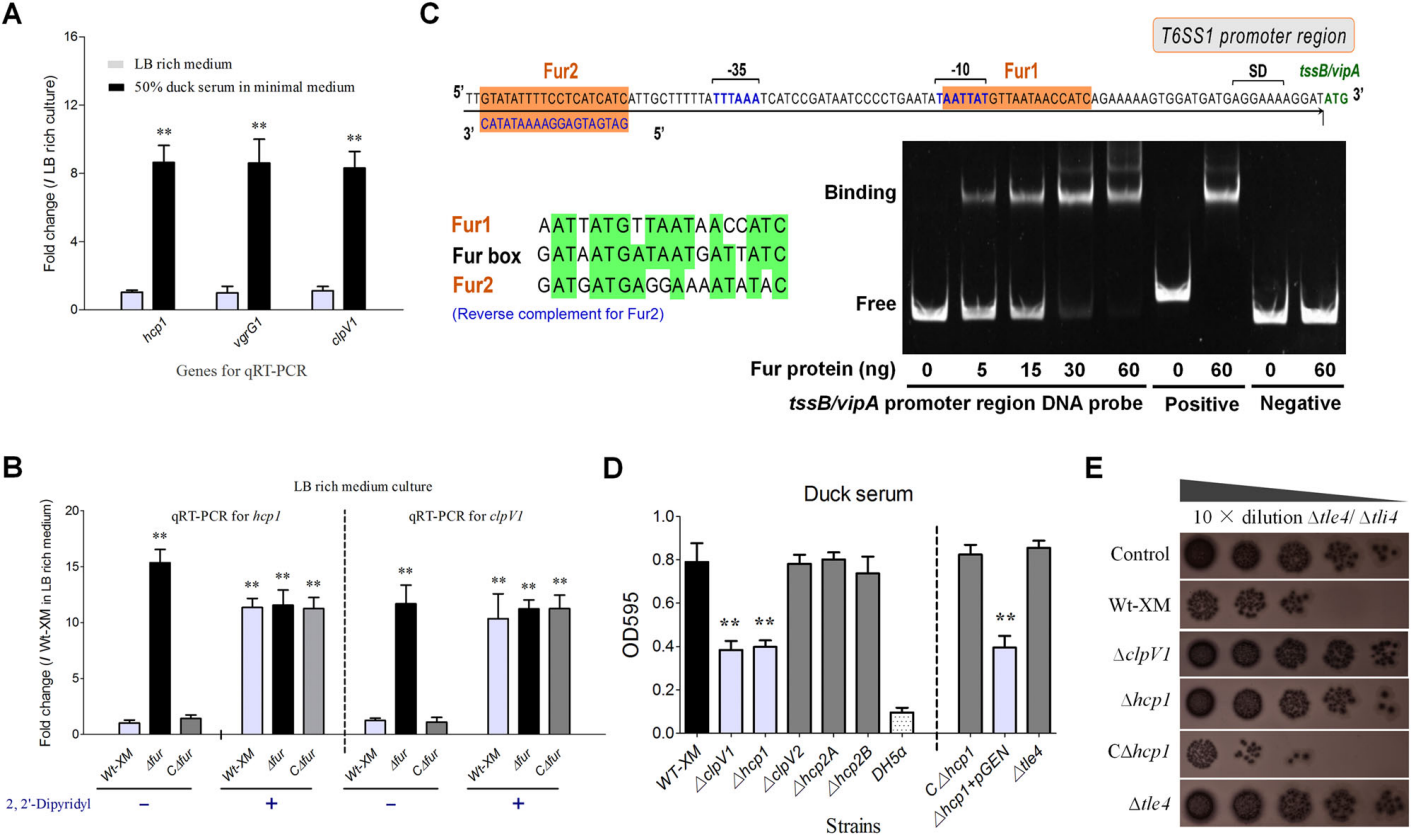
Transcriptional regulation and functional identification of the *hcp1* cluster. **a** The 50% duck serum in minimal medium could activate *hcp1* and *vgrG1* transcription. **b** The APEC T6SS1 gene cluster was regulated by the iron concentration and the Fur repressor. **a, b** Gene expression in the indicted conditions was analyzed by qRT-PCR. Expression levels were normalized against the level of the housekeeping gene *tus*. The relative expression levels are presented as the means ± SD obtained from three independently isolated RNA samples. **c** Fur directly regulated the T6SS1 operon. An *in silico* analysis was performed for the T6SS1 proximal promoter region. The ATG codon of *tssB*/*vipA* and the Shine-Dalgarno sequence (SD) are indicated. The putative −10 and −35 elements of the promoter (identified using the BProm program SoftBerry) are indicated in blue, and Fur-binding sequences are shown in orange boxes. In the nonradioactive EMSA assay of Fur, *fepA* promoter region DNA probes with and without Fur protein were used as positive controls, and *tssB*/*vipA* coding region DNA probes with and without the Fur protein were used as negative controls. **d** Hcp1 was involved in biofilm formation. The biofilm assay was performed using the 1% crystal violet method, **p < 0.01. Error bars represent the standard deviation for three independent experiments. **e** Hcp1 was required for the antibacterial activity of Tle4 against unprotected sister cells. The Tle4/Tli4 effector/immunity pair has been identified in our previous study^6^. The donor and recipient strains were mixed at a ratio of 3:1 and incubated for 6 h at 30 °C. Plate growth was demonstrated on Nal^R^ eosin-methylene blue agar, and the bacterial plaques (induced Nal^R^) represent increasing serial 10-fold dilutions from left to right

To explore the functional mechanisms of three Hcps, we managed to replenish the details of operon prediction and transcriptional analyses of the T6SS1 and T6SS2 clusters (Fig. 1b). The quantitative real-time PCR (qRT-PCR) results showed that the *hcp1-clpV1-vgrG1* cluster was significantly upregulated when the bacteria were cultured in M9 minimal medium with 50% duck sera (Fig. 2a). Fur has been identified as the main regulator of the increase in transcriptional activity of the *sci1*/T6SS1 cluster in response to iron limitation or acid exposure or in minimal medium in the enteroaggregative *E. coli* (EAEC) 042 strain^22^. In this study, inactivation of Fur or iron limitation could significantly activate the transcription of *hcp1* and *clpV1* in the APEC XM strain (Fig. 2b), suggesting that APEC isolates share a similar mechanism of T6SS1 regulation to that of the EAEC strain 042. However, there was no substantial sequence identity between the putative T6SS1 promoter region of the APEC XM strain and the *sci1*/T6SS1 promoter of the EAEC 042 strain^22^. In a reanalysis of the promoter sequence of T6SS1 using a bioinformatics approach, two Fur-binding sites were predicted (Fig. 2c). The Fur protein has been studied extensively as a repressor by blocking the promoter elements (−35 and −10 regions)^23, 24^. Once complexed to iron, Fur binds to a well-defined 19-bp sequence (5ʹ-GATAATGATAATCATTATC-3ʹ) called the ‘Fur box’^24, 25^. To determine whether Fur directly regulates T6SS1 gene expression, an electrophoretic mobility shift assay (EMSA) was performed. Indeed, the Fur protein was able to shift the promoter fragment of T6SS1 but not the negative control fragment (the coding region of *tssB*/*vipA*).

To validate whether the Fur-binding sites were necessary for the above-mentioned regulation, a point mutation was generated to modify the Fur-box sequence. DNA fragments of *vipAB*, including their native promoter and upstream regions of approximately 250 bp were amplified with site mutations in their Fur boxes to construct pGEN-vipAB* (Supplementary Table S2). Transcription levels of *vipA* were compared among four strains, that is, Δ*vipAB* and Δ*fur*Δ*vipAB* mutants with pGEN-*vipAB* or pGEN-vipAB*. The modification of the Fur-box sequence, similar to the deletion of *fur*, led to a significant increase in *vipA* expression in LB-rich medium (Supplementary Fig. S4). EMSA demonstrated that the DNA fragment with a modified Fur-box sequence could no longer be shifted by the Fur protein (Supplementary Fig. S4). These results suggested that Fur directly suppressed the expression of *hcp1* clusters in the iron replete condition.

### Hcp1-mediated biofilm formation and inter-bacterial competition

Previous studies have indicated that T6SSs are involved in biofilm formation in persistent infections, which contributes to enhanced bacterial pathogenicity^6, 26^. Our results showed that *hcp1* deletion caused significant attenuation of biofilm formation in 50% duck serum compared with the wild-type strain (*P < *0.01; Fig. 2d), and its complementation could restore this deficiency. These results were confirmed by tube cultures and scanning electron microscopy (SEM; Supplementary Fig. S5). However, Tle4, a known effector of T6SS1, was not involved in this phenotype (Fig. 2d), suggesting that Hcp1 contributed to biofilm formation via other unknown pathways. The antibacterial activity of the Tle4/Tli4 pair was markedly attenuated in the *hcp1* mutant, as determined by the visualization of prey cells in the selected plate (Fig. 2e), whereas *hcp1* complementation completely restored this deficiency, suggesting that Hcp1 plays a key role in the delivery of the Tle4 effector. Of note, a homolog of Tle4 with a PGAP1-like (post-glycosylphosphatidylinositol attachment to proteins1) domain has been reported to induce host autophagy by activating endoplasmic reticulum stress in *Pseudomonas aeruginosa*^17^, suggesting that Hcp1 is involved in pathogenicity via the delivery of the Tle4 effector.

### The *hcp2B* operon could be activated to function in inter-bacterial competition

**Fig. 3:**
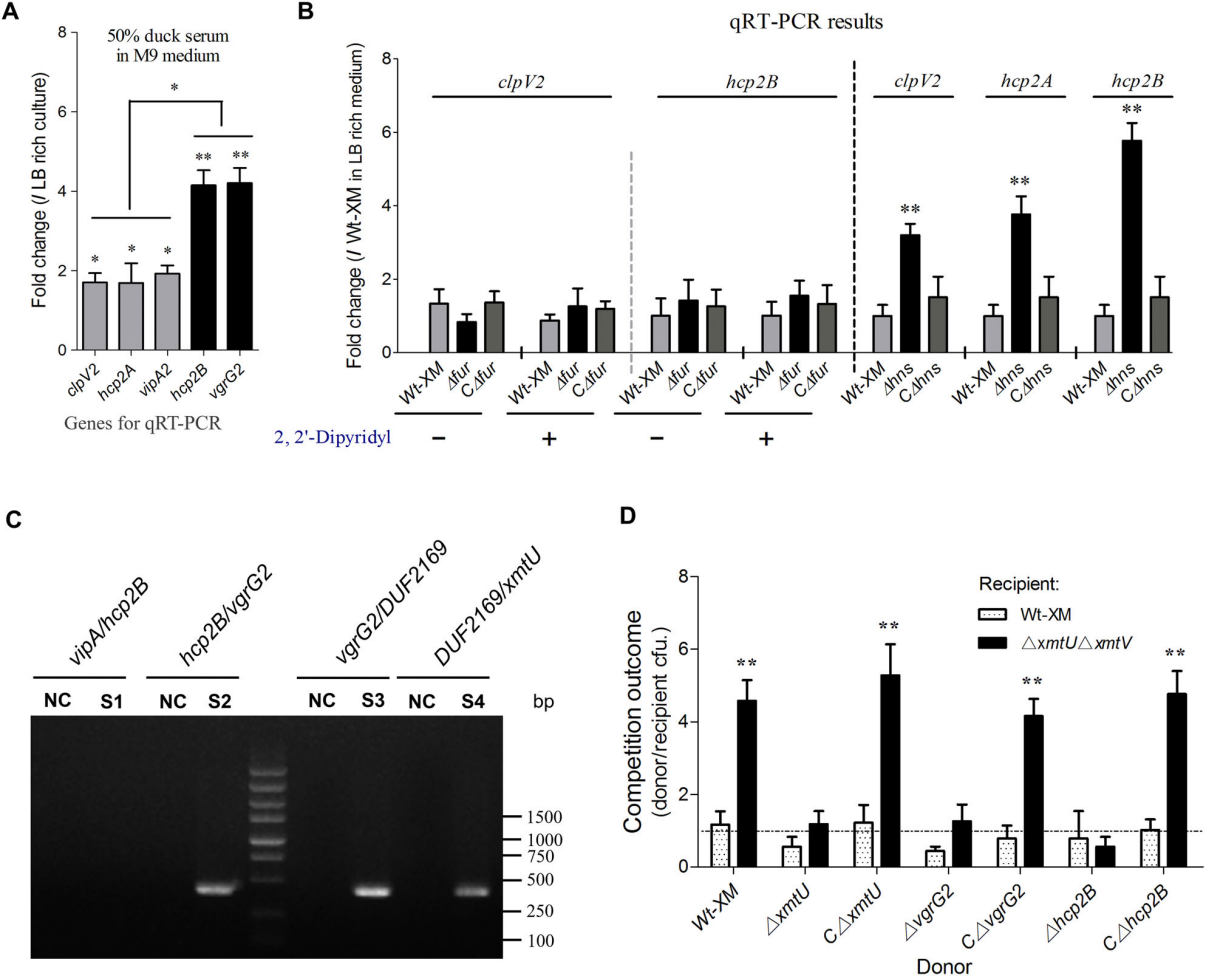
Transcriptional regulation and functional identification of the *hcp2B* and *hcp2A* clusters. **a** The 50% duck serum in minimal medium activated the transcription of *hcp2B-vgrG2* and *hcp2A-vipA* at different levels. **b** H-NS repressed transcription of the hcp2B cluster in LB-rich culture. The gene expression levels in the indicated conditions were analyzed by qRT-PCR. **c** Operon identification of *hcp2B-vgrG2*. The *hcp2B* and *vgrG2* genes formed one operon that deviated from the major cluster represented based on reverse transcription PCR, and a negative control was set up without reverse transcriptase. **d** Growth competition assays for the XmtU/XmtV pair between the indicated APEC donor and recipient strains. Experiments were initiated with equal CFUs of donor and recipient bacteria, as denoted by the dashed line. Asterisks indicate significant differences in competition outcomes between recipient strains against the same donor strain (**p < 0.01). Error bars indicate standard deviations for three independent experiments

Similar to the transcription of the *hcp1-vgrG1* cluster, the bacterial culture in minimal medium with 50% duck serum could also significantly upregulate the *vipA2-hcp2A* and *hcp2B-vgrG2* cluster (Fig. 3a). However, iron limitation and Fur repression did not regulate the transcription of *clpV2* and *hcp2B* (Fig. 3b). H-NS silences exogenous genes^27^, including T6SS clusters in *Salmonella enterica* and *Vibrio parahaemolyticus*^28, 29^. We found that the inactivation of H-NS could significantly upregulate the expression levels of *clpV2*, *hcp2A*, and *hcp2B* (Fig. 3b) but not *clpV1* and *hcp1* (Supplementary Fig. S6). Similar results have been obtained for the T6SS2 cluster of the enterohemorrhagic *E. coli* strain EDL933^30^, but the mechanism underlying the relief of H-NS repression has not been determined. Anaerobic culture, osmotic pressure, and temperature changes were evaluated to determine the potential regulatory pathways, and no significant differences were observed in the transcription levels of *clpV2*, *hcp2A*, and *hcp2B*. Notably, *hcp2B-vgrG2* and *vipA2-hcp2A* from the T6SS2 cluster displayed differences in regulation in 50% duck serum (Fig. 3a), and they were transcribed in the opposite direction (Fig. 1b), suggesting that the orphan *hcp2B*-*vgrG2* might be regulated by a specific mechanism.

It is reasonable to infer that *hcp2B*-*vgrG2* is controlled by different operons from the major cluster of T6SS2. Prediction by the BProm program SoftBerry followed by reverse transcription PCR indicated that *hcp2B*-*xmtV* forms one operon (Fig. 3c) and thus shares a single promoter. A homolog of the DUF2169 protein in the hcp2B operon (Fig. 1b) has been identified as an adaptor/chaperone protein of the T6SS antibacterial effector^31, 32^, suggesting that downstream genes encode an effector/immunity pair to mediate inter-bacterial antagonism. Using the structural prediction program HHpred, XmtU was demonstrated to contain an N-terminal PAAR-repeat motif^33^ and a C-terminal peptidase_C39 toxin domain^34^ (Fig. 1b), suggesting that this toxic enzyme is a potential antibacterial effector. Previously, characterized T6SS-dependent toxic effectors consistently coexist with antagonistic immunity proteins encoded by downstream genes;^35^ thus, we predicted that *xmtV* is the cognate immunity gene of *xmtU*. Indeed, a double knockout mutant lacking both *xmtU* and *xmtV* was efficiently reduced by fivefold when exposed to TW-XM or CΔ*xmtU* (Fig. 3d), and protein binding between XmtU and XmtV was confirmed by a pull-down assay (Supplementary Fig. S7), indicating that XmtU/XmtV was a functional T6SS-related effector/immunity pair. Additionally, the antibacterial activity of XmtU could be completely abolished by inactivation of Hcp2B or VgrG2 (Fig. 3d), indicating that these two proteins were critical for XmtU delivery. These findings suggested that the *hcp2B* operon could be activated to function in inter-bacterial competition.

### Structural comparison of Hcps indicated diversity in the extended loop regions

**Fig. 4:**
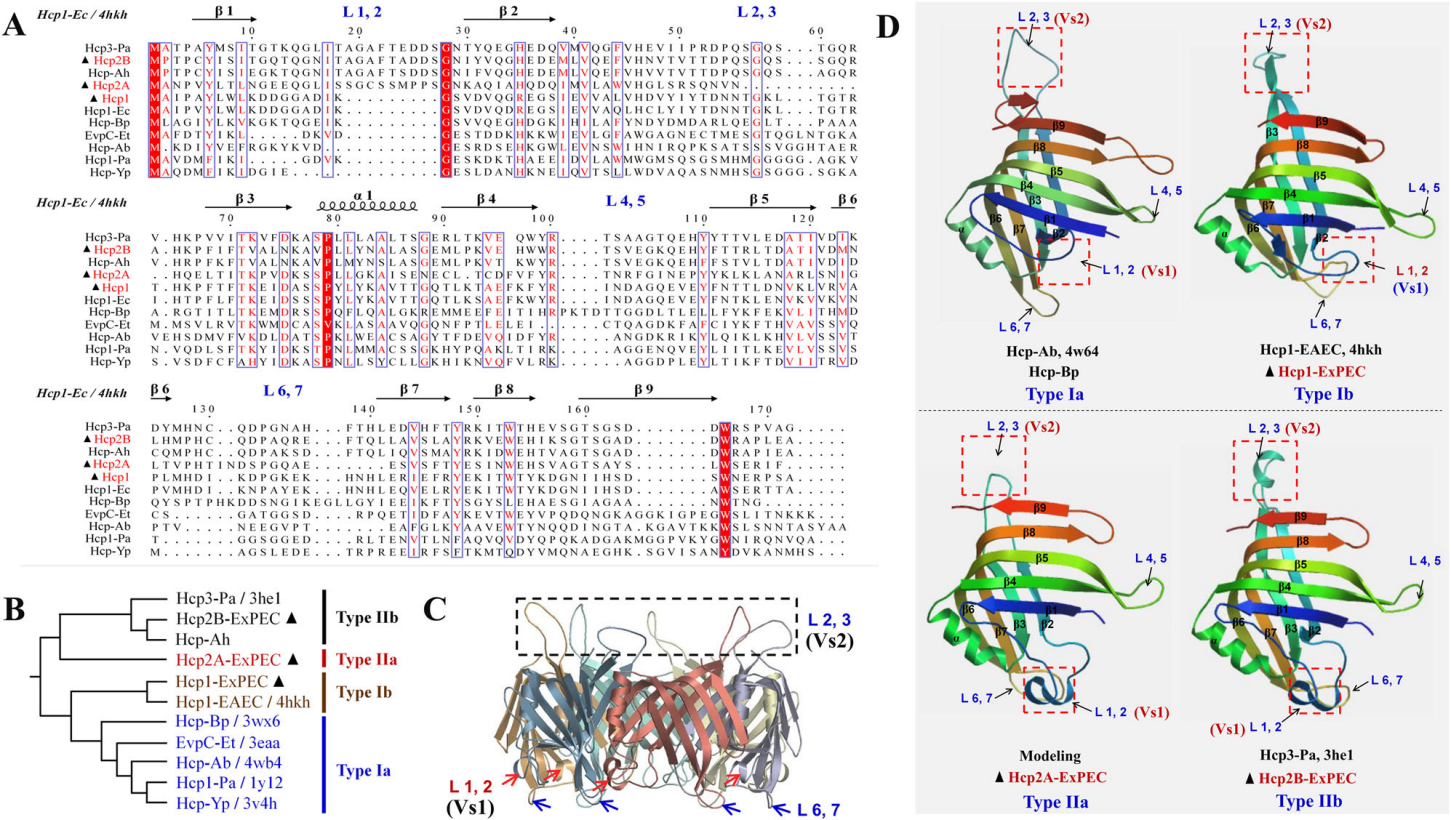
Sequence alignment and structural comparison of four distinct Hcp types. **a** Sequence alignment and secondary structure of ExPEC Hcp proteins with other known structural homologs. The alignment of Hcp amino-acid sequences was obtained using ClustalW. **b** The phylogenetic tree and types of 11 Hcp family members selected from Fig. 1a. **c** The Hcp hexamer of Hcp3 from *Pseudomonas aeruginosa* (PDB code: 3he1). **d** Structural comparison of four distinct Hcp types. Hcp-Bp (PDB code: 4w64) is referred to as type Ia, Hcp1-EAEC (PDB code: 4hkh) is referred to as type Ib, and Hcp3-Pa (PDB code: 3he1) is referred to as type IIb. The structural model of Hcp2A (from ExPEC) was drawn based on the template 3he1 (sequence identity, 43.05%) using the SWISS-MODEL server and is referred to as type IIa. The L1, 2 (Ala19 to Ser27) and L2, 3 (Ser56 to His66) loops were designated Variant sequence 1 (Vs1) and Vs2 regions, respectively

The Hcp protein is a core component of the T6SS nanomachine, and its deletion results in a non-functional secretion apparatus. The effects of Hcps are more likely due to its control of the secretion process of other effector proteins rather than to a direct effect of Hcps itself. However, several T6SS clusters encode more than one *hcp* gene, exhibiting differences in transcription, assembly, and function^7, 36^. Thus, we speculated that the specific regulation of *hcp* promoters and minor modifications of the Hcp protein structure explain its specific properties. Currently, eight crystal structures of Hcp proteins are available (Figs. 1a, 4a), and these can be divided into four genetic types (Figs. 1a, 4b). All these Hcp homologs present limited sequence identities ranging from 15 to 60% (Fig. 4a), whereas their secondary structures are highly conserved and very similar overall (Figs. 4c, d)^21, 37^. Despite the close matching of the backbones of tertiary structures, the loops exhibited significant differences, especially the overhang loops L1, 2 (Ala19 to Ser27) and L2, 3 (Ser56 to His66; Fig. 4). In this study, Hcp1, Hcp2A, and Hcp2B from APEC belonged to Type Ib, IIa, and IIb, respectively (Fig. 4b). Similarly, their sequence alignments indicated significant discrepancies within loops L1, 2 and L2, 3 (Figs. 4a, d), which were designated Variant sequence 1 (Vs1) and Vs2, respectively.

### The Vs2 region was essential for the delivery of antibacterial effectors

**Fig. 5:**
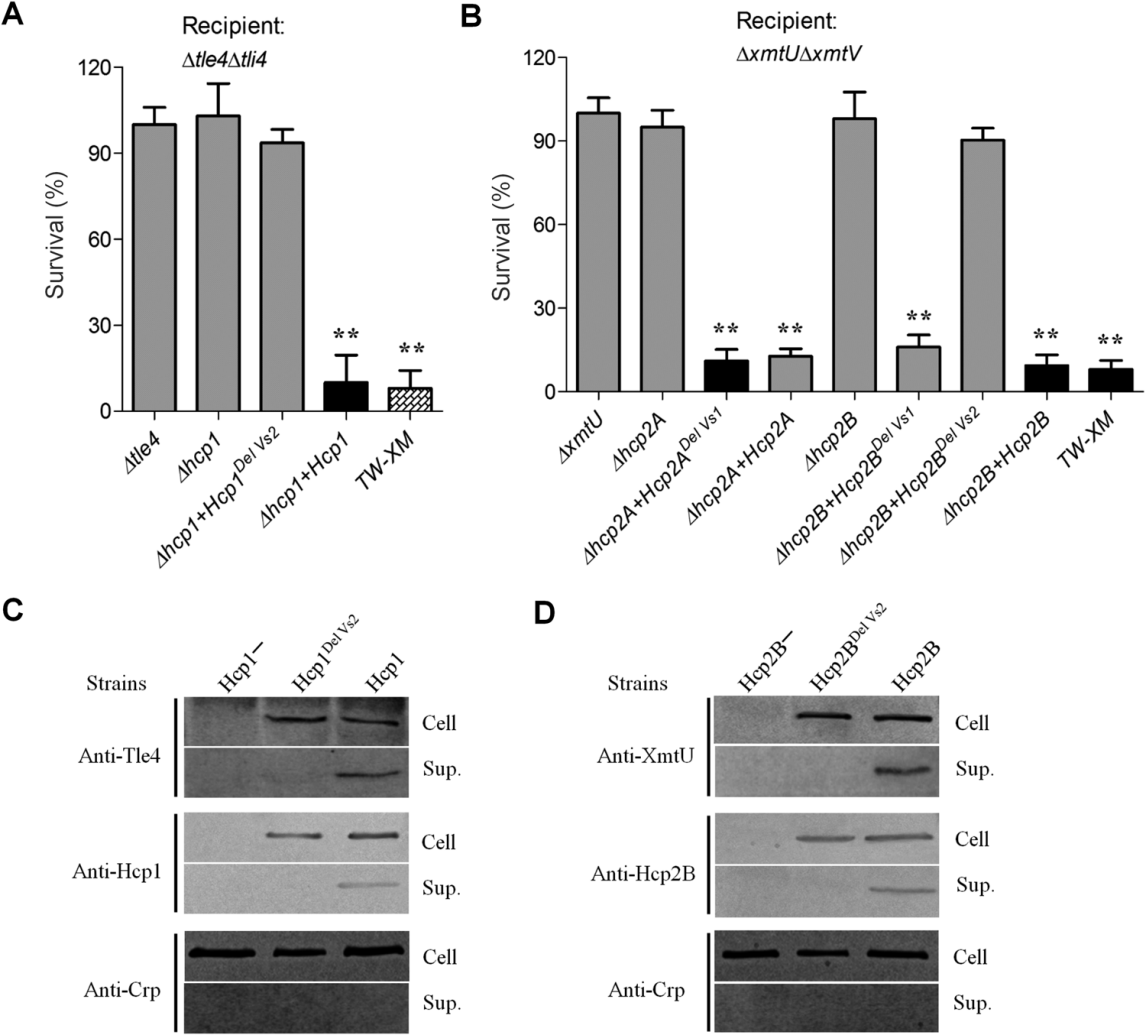
Deletion of the Vs2 region in Hcp1 and Hcp2B impaired the secretion of the corresponding antibacterial effectors. Antibacterial capability was completely abolished in Tle4- or XmtU-positive donors, suggesting the deficiency of Hcp tube assembly for effector secretion. The plasmid pGEN-MCS was used to express *hcp1*, *hcp1*^*DelVs2*^, *hcp2A*, *hcp2A*^*DelVs1*^, *hcp2B*, *hcp2B*^*DelVs1*^, and *hcp2B*^*DelVs2*^. Error bars represent the standard deviations from three independent experiments. Asterisks denote competitive outcomes that are significantly different from those obtained with Δ*tle4* or Δ*xmtU* (**p < 0.05). **a** Outcome of growth competition between the indicated donor strains on the *x*-axis and Δ*tle4*Δ*tli4*. **b** Growth competition assay for the indicated donor strains on the *x*-axis and Δ*xmtU*Δ*xmtV*. **c** Deletion of Vs2 in Hcp1 impaired effector secretion without affecting Tle4 stability in the cytoplasm. **d** Vs2 of Hcp2B was required for effector secretion but was not involved in XmtU stability in the cytoplasm. Crp (cyclic AMP receptor protein) is a cytoplasmic protein marker

A recent study has shown that mutation of the two residues within loop L2, 3 could abolish the stacking of Hcp hexamers to form tubular structures in *Burkholderia pseudomallei* and impair the secretion of effectors^12^. Herein, deletion of the Vs2 region (within loop L2, 3) in Hcp1 of APEC also completely abolished the antibacterial capability of Tle4 against the mutant Δ*tle4*Δ*tli4* (Fig. 5a). Similarly, the donor strain producing the Vs2-lacking Hcp2B variant also lost its competitive advantage with respect to the XmtU toxin over the recipient strain Δ*xmtU*Δ*xmtV* (Fig. 5b). Notably, a previous study has demonstrated that Hcp proteins can promote the stability of antibacterial effectors within the cytoplasm via specific interactions^9^. Thus, we assessed the effect of the Vs2 deletion in Hcps on the stability or secretion of antibacterial effectors. The deletion of Vs2 in Hcps did not affect the stability of Tle4 or XmtU in the cytoplasm (Figs. 5c, d), indicating that the interactions between Hcp^DelVs2^ and effectors functioned properly. Notably, the Vs2 deletion completely interdicted the secretion of Hcp1^DelVs2^, Hcp2B^DelVs2^, Tle4 and XmtU (Figs. 5c, d), suggesting that the Hcp^DelVs2^ hexamers were insufficient to pack tubular structures for effector translocation. However, the Vs1 deletion in Hcp2A and Hcp2B had no effect on the antibacterial capability against the recipient Δ*xmtU*Δ*xmtV* (Fig. 5b), and its function thus remained unclear.

### The Vs2 region was required for inhibition of macrophage phagocytosis

**Fig. 6:**
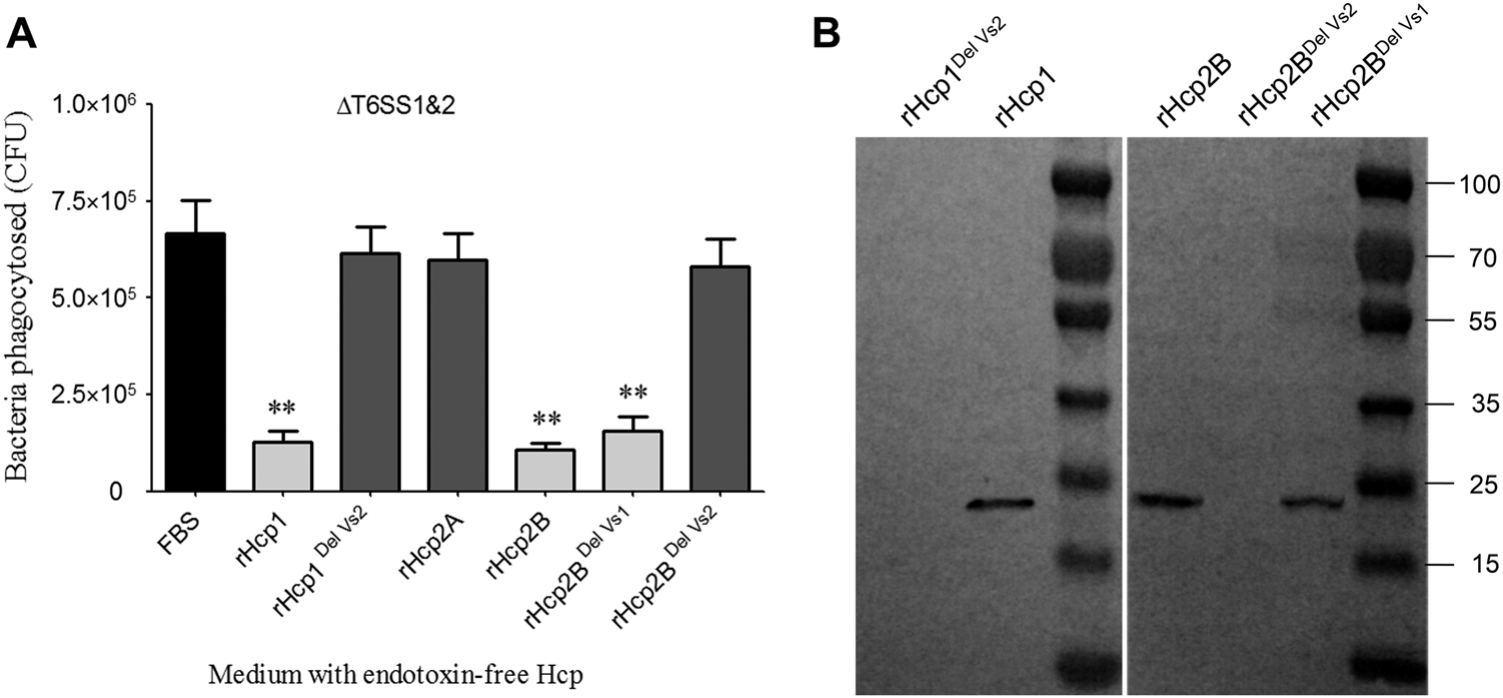
Hcps bound to macrophages and inhibited phagocytosis. **a** The endotoxin-free rHcp1 and rHcp2B could significantly inhibit phagocytosis of RAW 264.7 cells. The functions of T6SS1 and T6SS2 were deficient in Δ*clpV1*Δ*clpV2*, which could avoid the effects of other T6SS effectors. Error bars represent the standard deviations from three independent experiments, **p < 0.01. **b** The Vs2 region played a key role in rHcp1 and rHcp2B binding to macrophages. Endotoxin-free rHcps (25 μg) was co-incubated with RAW 264.7 cells for 4 h and washed five times with MEM

The secreted Hcp rings can be inserted into the eukaryotic membrane as part of the “translocon”^8^ and plays a possible role in evasion from host innate immunity^14, 38^. The inhibition of macrophage phagocytosis by the Hcp protein has been confirmed in *Aeromonas hydrophila*^38^, but it has not been evaluated in *E. coli*. Significant attenuation of macrophage phagocytosis was detected after co-incubation of 25 μg of endotoxin-free rHcp1 (Hcp1_His6_) or rHcp2B (Hcp2B_His6_) with RAW 264.7 cells for 4 h (Fig. 6a). rHcp1 or rHcp2B was detected in the membrane fractions of co-incubated RAW 264.7 cells. Furthermore, variants of rHcp1 or rHcp2B carrying the deletion of the Vs2 region exhibited complete inactivation of binding to the cytomembrane of macrophages (Fig. 6b), suggesting that the Vs2 region played an essential role in the inhibition of macrophage phagocytosis. As shown in Figs. 5c, d, Hcp^DelVs2^ may be defective in the formation of stabilized hexamers and in packing tubular structures for effector translocation. Thus, it is possible that the large hexamers or tubes formed by native Hcp are more easily attached to macrophage cells than Hcp^DelVs2^ monomers or unstabilized hexamers. However, the Vs1 region was dispensable for this process, which may explain why rHcp2A (only containing the Vs1 region) could not impair macrophage phagocytosis (Fig. 6a).

### Hcps were involved in immunoprotection in the duck model

**Fig. 7:**
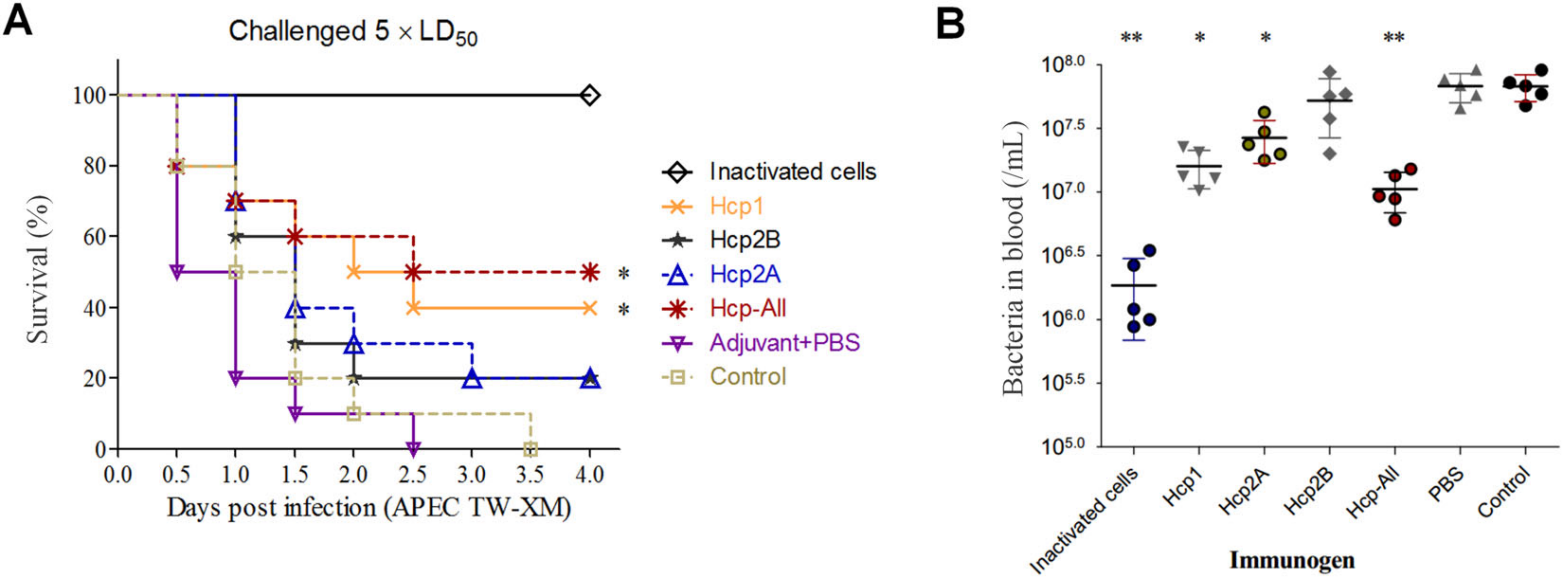
rHcps provided partial immunoprotection in the duck model. **a** Summary of the immunoprotection assays used for rHcp proteins in the duck model. Control groups were immunized with the flow-through samples of the Ni-NTA Spin Column (QIAGEN) from the empty BL21 lysate. The anti-Hcps serum titers of ducks were determined by ELISA (Supplementary Fig. S3) prior to challenge with APEC TW-XM at 1 × 10^6^ CFU per duck. Survival data were analyzed by the Kaplan–Meier Estimator method based on comparisons with the control group, *p < 0.05, **p < 0.01. **b** *In vivo* infection studies. Ducks immunized with rHcps (Hcp_His6_ proteins) were intraperitoneally infected with lethal doses (~5 × LD_50_) of bacteria. Reisolation of APEC from the blood was performed, and bacteria were quantified by plate counting at 24 h post-inoculation. Error bars indicate the standard deviations of recovered bacteria from the blood for each group. Statistical significance was determined by Student’s *t-*test based on comparisons with the control group (**p < 0.01, *p < 0.05)

Recently, two Hcps of NMEC RS218 were shown to induce actin cytoskeleton rearrangement, apoptosis, or the release of IL-6 and IL-8 in human brain microvascular endothelial cells, indicating the important roles of Hcp in bacteria–host interactions^7^. Notably, these two Hcps share highly similar amino-acid sequences (identity, >99%) with Hcp2A and Hcp2B of APEC (Supplementary Fig. S8). More importantly, infected ducks might produce specific antibodies against Hcp1, Hcp2A, and Hcp2B to abolish the pathogenic functions of T6SSs (Fig. 1b), suggesting that these Hcps have potential as promising vaccine candidates to prevent ExPEC infection. Thus, purified rHcps were used to vaccinate ducks (Supplementary Fig. S2), and the antigen-specific humoral response was confirmed (Supplementary Fig. S3). Subsequently, the survival rate was assessed after challenge with a lethal dose of APEC (~5 × LD_50_). Indeed, the group immunized with Hcp-All (all three Hcps) showed the highest survival rate (50%), followed by the Hcp1 group (40%; Fig. 7a). To further verify these results, ducks were challenged to determine the effect of Hcp immunization on the colonizing ability of bacteria in the blood by plate counting. As shown in Fig. 7b, bacterial colonization was significantly reduced in groups vaccinated with Hcps-all, Hcp1, or inactivated APEC cells. These observations verified that anti-Hcps antibodies offered partial protection against APEC infection in the duck model. However, there were distinct differences among groups immunized with different rHcps, suggesting that these Hcps might play dissimilar roles in the infection process.

## Discussion

T6SS is involved in a wide range of processes, including inter-bacterial competition, stress sensing, biofilm formation, and host pathogenesis^26, 39^. To enable such diverse functions of T6SS, precise regulatory modulations are needed^36, 40^. Indeed, a wide array of different mechanisms have been reported, for example, control by histone-like proteins (H-NS)^28^, transcription factors (Fur)^22^, quorum-sensing mechanisms^41^, or secondary-messenger molecules (ppGpp)^42^. Most T6SSs need to be activated by low temperatures, iron starvation, host tissues/extracts, human plasma, or macrophages in *Yersinia pestis*, *Vibrio cholera*, *Salmonella enteric*, *Francisella tularensis*, and *Burkholderia pseudomallei*^36^. In this study, we confirmed that the *hcp1* and *hcp2B* clusters were repressed by Fur and H-NS regulators in LB-rich medium, respectively, and bacterial cultures in minimal medium with 50% duck serum exhibited significantly enhanced transcription levels. Further analyses revealed that iron-depleted conditions in duck serum relieved Fur-mediated repression of the *hcp1* cluster, which mediated biofilm formation and inter-bacterial competition. However, the intrinsic mechanism underlying H-NS derepression in *hcp2B* clusters is still unknown. Thus, these Hcps are core components of T6SS complexes in APEC, contributing to inter-bacterial competition, biofilm formation in serum, and immunoprotection against lethal challenges. By hijacking specific physical or chemical signals, bacteria “recognized” their localized microenvironment to regulate the T6SS assembly for pathogenic or competitive purposes, which further contributes to maintaining adaptive survival in ecological niches.

Hcp is essential for both the assembly of T6SS and the export of its effectors^21, 38^, and it has been identified as a chaperone, substrate receptor, and secreted protein^9^. In this study, the subset of Hcps from Gram-negative bacteria was first divided into four distinct types according to a phylogenetic analysis. Although many bacterial genomes encode more than one Hcp protein, these loci were not functionally redundant, as in *Burkholderia thailandensis*; Hcp-1 mediates bacterial antagonism, whereas Hcp-5 promotes intercellular spread in infected macrophages^26^. Similar observations also have been reported in *Pseudomonas aeruginosa*^16^. Indeed, Hcps not only interacts with VgrG trimers from different T6SS complexes^43^, but they also bind to or fuse with diverse effector domains^9, 44^, and these multiple functions may drive the bacterial cells to acquire additional Hcp homologs to promote the functional differentiation of T6SSs. These findings may partially explain why three different types of Hcps are present in a single strain. Although these Hcp homologs shared limited amino-acid sequence identities, their secondary structures were well conserved and their overall tertiary models were very similar. However, the Hcp monomer still exhibited structural differences with some delicate modifications, suggesting that the characterization of Hcp types will help to clarify the discrepancy in their properties. Two variable regions, Vs1 (in Loop L1, 2) and Vs2 (in Loop L2, 3), were identified in this study, and further study confirmed that deletion of the Vs2 region in Hcp1 and Hcp2B caused a failure of effector secretion, inhibition of phagocytosis, and binding to macrophages.

It should be noted that Hcp1 and Hcp2B modules delivered different effectors, that is, Tle4 and XmtU, respectively. A homolog of Tle4 with a PGAP1-like domain has been reported to induce host autophagy by activating endoplasmic reticulum stress in *Pseudomonas aeruginosa*^17^, suggesting that Hcp1 is involved in pathogenicity via delivery of the Tle4 effector. Many peptidase effectors (including Tae family, PAAR-Rhs-MPTase4) mediate antibacterial effects^45, 46^. Notably, two peptidase families, M35 and M60 zinc-metalloproteases, are associated with bacterial pathogenicity^47, 48^. In the type III secretion system (T3SS), two zinc-metalloprotease effectors, NleC and NleD, are involved in intestinal pathogenicity by blocking nuclearfactor-kappaB (NF-κB) and activator protein 1 (AP-1) activation, respectively^49^. XmtU is a member of the Peptidase_C39 family, suggesting that XmtU also has the potential to facilitate APEC virulence in the pathogenic process, but this phenomenon requires further exploration.

T6SS tubes formed by the stacking of Hcp hexamers have been identified in different bacteria, but the precise roles of Hcps in interactions with host cells remain to be clarified. Although Hcp homologs in *Aeromonas hydrophila* and *Burkholderia pseudomallei* have been shown to modulate the activation of host immune cells^12, 14^, the molecular mechanisms in other pathogens have not been explored. The three Hcps in T6SS1 and T6SS2 (Supplementary Fig. S8) are critical for ExPEC pathogenicity in systematic or cerebral infection^7, 50^. The competitive advantage, biofilm formation, and phagocytosis inhibition mediated by Hcps and their effectors are beneficial for bacterial colonization, proliferation, and persistent infections during the pathogenic process^6, 26^. In this study, the effective immunoprotection in the group immunized with three Hcps may occur by abolishing these advantages. Although a limited protective efficacy was obtained by immunization with recombinant Hcp1, Hcp2A, or Hcp2B against APEC infection in the duck model, further optimization of adjuvants and immunization strategies should be evaluated for the development of vaccines or serodiagnostic markers.

In conclusion, three distinct Hcp types in ExPEC (Supplementary Fig. S8) exhibited differences in transcriptional regulation and were involved in pathogenicity, inter-bacterial competition, or biofilm formation in host serum. The deletion of Vs2 in Hcp1 and Hcp2B significantly impaired interactions with host cells and the export of the antibacterial effectors Tle4 and XmtU, respectively. In short, the functional differentiation of Hcp homologs is driven substantially by specific transcriptional regulatory mechanisms, diverse extended loop regions, and effector delivery.

## Materials and methods

### Ethics statement and animals

All animal experiments were carried out according to animal welfare standards and approved by the Department of Science and Technology of Jiangsu Province. The license number was SYXK (SU) 2010-0005. Seven-day-old ducks were used for the challenge with APEC strains and for immunoprotection analyses of Hcp proteins.

### Bacterial strains and growth conditions

The bacterial strains used in this study are listed in Supplementary Table S1. The APEC XM (O2: K1) isolate is the model strain used for T6SS research in our lab^50^ and can cause serious meningitis in duck and neonatal rats. The *E. coli* strain BL21 (DE3) (Invitrogen, Carlsbad, CA, USA) was used for protein expression. All *E. coli* strains were cultured in Luria–Bertani broth (Oxoid, Hampshire, UK) at 37 °C with aeration, unless otherwise indicated. When necessary, Luria-Bertani (LB) broth was supplemented with kanamycin (Kan, 50 µg/mL), ampicillin (Amp, 100 µg/mL), chloramphenicol (Clm, 25 µg/mL), or nalidixic acid (Nal, 50 µg/mL).

### Phylogenetic and prevalence analyses

Phylogenetic analyses were performed following the procedures outlined by Bingle et al^51^. A ClustalW alignment was generated using the Hcp amino-acid sequences. A phylogenetic tree was constructed using MEGA (v.5.0.3) with the neighbor-joining method with Poisson correction and bootstrapping (*N *= 1000 replicates).

### Analysis of gene expression levels by real-time PCR

Bacteria were incubated in LB medium or M9 minimal medium with 50% duck serum, and RNA was isolated using the E.Z.N.A. Bacterial RNA Isolation Kit (Omega, Norcross, GA, USA). Residual genomic DNA was removed using DNase I (New England Biolabs, Ipswich, MA, USA), and complementary DNA synthesis was performed using the PrimeScript RT Reagent Kit (Takara, Shiga, Japan). qRT-PCR was performed to determine the transcription levels of the T6SS core genes using SYBR Premix Ex *Taq* (TaKaRa) and gene-specific primers (Supplementary Table S2), and the data were normalized to the levels of the housekeeping gene *tus*^52^. The relative fold change was calculated using the threshold cycle (2^-△△*CT*^) method^53^. Values are reported as the means plus standard deviations (error bars) of three independent RNA extractions.

### Expression and purification of recombinant proteins

The construction of recombinant plasmids (primers in Supplementary Table S2) and protein expression were performed following standard molecular cloning procedures. The His_6_ fusion proteins were purified twice using Ni-NTA Spin Columns (QIAGEN, Hilden, Germany) and then ultrafiltered using 10.0-kD cutoff spin columns (Millipore, Burlington, MA, USA) to maintain homogeneity. The purified recombinant proteins were used to perform immunoprotection assays or for the preparation of rabbit immune sera of three Hcps by Zoonbio Biotechnology Co. Ltd (Nanjing, China).

### Construction of mutants

Disruptions of *hcp* genes in the APEC XM strain (Δ*hcp1*, Δ*hcp2A*, and Δ*hcp2B*) were obtained by λ Red mutagenesis^54^. The plasmids pGEN-*hcp1*^*Del Vs2*^ and pGEN-*hcp2B*^*Del Vs2*^ were constructed as described in Supplementary Table S1 and then transferred into Δ*hcp1* and Δ*hcp2B* to achieve the Vs2 deletion of Hcp1 and Hcp2B, respectively. Similarly, pGEN-*hcp2A*^*Del Vs1*^ and pGEN-*hcp2B*^*Del Vs1*^ were transferred into Δ*hcp2A* and Δ*hcp2B* to achieve the Vs1 deletion, respectively. The Variant sequence 1 (Vs1) and Vs2 regions were identified as Ala19–Ser27 and Ser56–His66 in the sequence alignment of 11 Hcps, respectively. All primers used in this study are listed in Supplementary Table S2.

### Inter-bacterial competition and recipient survival assays

Inter-bacterial antagonism assays were performed as described previously^6^. Briefly, both strains were adjusted to an OD_600nm_ of 0.5 and mixed at a 1:1 ratio (donor to recipient) in triplicate. This mixture (2.5 µL) was then spotted on a 2.5% agar LB low-salt plate with a 0.2-µm nitrocellulose membrane for 6 h of incubation at 30 °C. Bacterial spots were then serially diluted in phosphate-buffered saline and visualized on LB plates containing 40 µg/mL X-gal and 0.1 mM IPTG to distinguish donor (*lacZ*^+^) and recipient (*lacZ*^−^) colonies via blue/white screening. The spontaneous nalidixic acid-resistant (Nal^R^) mutants of recipient strains were screened by plating 10^9^ colony-forming unit (CFU) onto LB containing 50 µg/mL Nal and confirmed to retain the full growth properties^55^.

### Electrophoretic mobility shift assay

*E. coli* BL21 with pET21a-*fur* was grown in 100 mL of LB medium for 20 h at 16 °C, and protein expression was induced by addition of 0.1 mM IPTG. The Fur protein was purified by ion-metal affinity chromatography as previously described^56, 57^. DNA probes were amplified using specific primers and purified using a Takara MiniBEST Gel Extraction Kit. PCR products were incubated in a final volume of 25 μL in Fur EMSA binding buffer (10 mM Tris-borate, 40 mM KCl, 7.5% glycerol, 1 mM MgCl_2_, 100 μM MnCl_2_, 4 mM DTT, 100 μg/mL bovine serum albumin, and 1 μg/mL sonicated salmon sperm DNA, pH 7.5) at 5 ng with increasing concentrations of the Fur protein at 37 °C for 30 min as previously described^22^. The reaction mixtures were then subjected to electrophoresis on a 6% polyacrylamide gel in Tris-borate buffer (45 mM Tris base, 45 mM boric acid, 100 μM MnCl_2_ buffer) at 200 V for 45 min. The gel was stained in the above electrophoretic buffer containing 1 × SYBR Green Nucleic Acid Staining Solution (Life Technologies, Carlsbad, CA, USA) for 30 min, and images were recorded.

### Anti-phagocytosis and Hcp-binding assay

Phagocytosis assays were performed as described previously^58^ with some modifications. RAW 264.7 cells were pre-incubated in the presence or absence of 25 μg of endotoxin-free rHcps (Hcp_His6_ proteins) for 4 h and then infected with 5 × 10^6^ CFU ΔT6SS1&2 strains at a multiplicity of infection (MOI) of 1:50 for 1 h (in minimum Eagle’s medium (MEM) without fetal bovine serum). Then, the cells were washed and treated with Dulbecco’s modified Eagle’s medium containing gentamicin (100 μg/mL) for 1 h to kill extracellular bacteria. The host cells were lysed with 1 mL of sterile water for 20 min (for complete lysis), and the cell lysates were counted on LB agar plates to determine the CFU. Values are reported as the means plus standard deviations (error bars) of three independent experiments. The residual samples were centrifuged to obtain a pellet as the membrane fraction of RAW 264.7 cells and tested for the presence of rHcp using anti-His monoclonal antibodies (Invitrogen) by immunoblotting analysis. Blots were developed using ECL plus reagent (GE Healthcare, Little Chalfont, UK).

### Biofilm formation assay and SEM

The biofilm assay (1% crystal violet method) was performed as previously described in 96-well plates with duck serum^6^, and *A*_595_ was recorded. Wells with the DH5α strain cultured in 50% sterile duck serum served as controls. All biofilm assays were run in triplicate. SEM was performed as previously described^6^. Observations were performed at 15 kV with a scanning electron microscope (model S800; Hitachi, Tokyo, Japan).

### Immunization and challenge studies

The 7-day-old ducks were divided into seven groups of 16 ducks each. Each group was inoculated twice with 50 µg of the corresponding immunogen per duck by subcutaneous injection in the back at an interval of 10 days. Control groups were inoculated with the flow-through sample from the Ni-NTA Spin Columns (QIAGEN) from the empty BL21 lysate. Serum titers were evaluated by indirect ELISA to confirm the production of the IgG antibody^59^. Subsequently, animals were challenged intraperitoneally with the APEC strain TW-XM (1 × 10^6^ CFU per uck). The groups were observed over a 7-day period, and survival was recorded every 12 h. Furthermore, the colonization capacity of the TW-XM strain in immunized ducks was measured at 24 h post-infection by euthanasia and autopsy. The bacterial quantity was determined by tissue homogenization, appropriate dilutions, and plate counts on MacConkey agar.

### Hcp structural comparison

A structural comparison of the four Hcp types was performed. An alignment of Hcp amino-acid sequences was obtained using ClustalW. Crystal structures of Hcp-Bp (4w64, type Ia), Hcp1-EAEC (4hkh type Ib), and Hcp3-Pa (3he1, type IIb) were obtained from published data in the NCBI PDB database. The structural model of Hcp2A (from ExPEC, type IIa) was drawn based on the template 3he1 (sequence identity, 43.05%) using the SWISS-MODEL server (http://swissmodel.expasy.org). The Hcp hexamer structure for 3he1 was evaluated to more clearly observe the extended loops^37^.

### Statistical analysis

Statistical analyses for *in vitro* and *in vivo* experiments were implemented in Prism 5 (GraphPad Software, La Jolla, CA, USA). One-way analysis of variance (ANOVA) was used for the anti-phagocytosis assays, and two-way ANOVA was used to evaluate the qRT-PCR and biofilm formation results. The duck challenge and survival data were analyzed by the Kaplan–Meier method and the log-rank test.

## Electronic supplementary material

Supplementary Figure S1

Supplementary Figure S2

Supplementary Figure S3

Supplementary Figure S4

Supplementary Figure S5

Supplementary Figure S6

Supplementary Figure S7

Supplementary Figure S8

Supplementary Table S1

Supplementary Table S2

Supplementary data
