# Supplementary material for: Three Hcp homologs with divergent extended loop regions exhibit different functions in avian pathogenic Escherichia coli
Source: Emerg Microbes Infect. 2018 Mar 29;7:49. doi: 10.1038/s41426-018-0042-0 (PMC5874247; doi:10.1038/s41426-018-0042-0)
Supplement: Supplementary file 1 — Supplementary Figure S1 [file 41426_2018_42_MOESM1_ESM.docx]

**
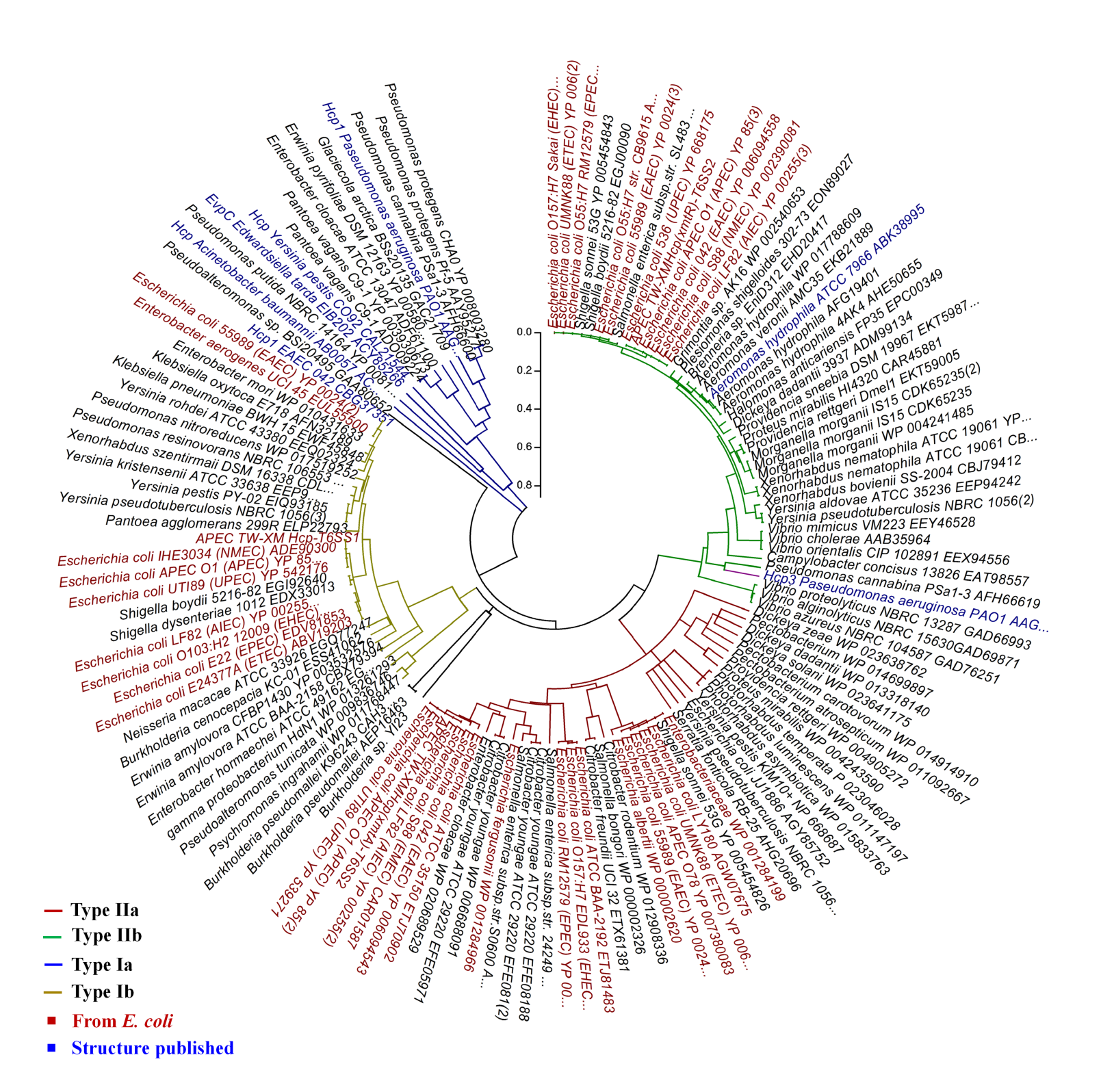
**

**Figure S1 Evolutionary relationships among 136 known and putative Hcps from gram-negative bacteria.** A neighbor-joining tree (1000 bootstrap replicates; Poisson correction) was constructed based on a ClustalW alignment of the Hcp amino acid sequences. Hcps from *E. coli* strains are indicated in red. The four types of Hcp are indicated by red, blue, green, and khaki branches.
