# Supplementary material for: Three Hcp homologs with divergent extended loop regions exhibit different functions in avian pathogenic Escherichia coli
Source: Emerg Microbes Infect. 2018 Mar 29;7:49. doi: 10.1038/s41426-018-0042-0 (PMC5874247; doi:10.1038/s41426-018-0042-0)
Supplement: Supplementary file 2 — Supplementary Figure S2 [file 41426_2018_42_MOESM2_ESM.docx]

**
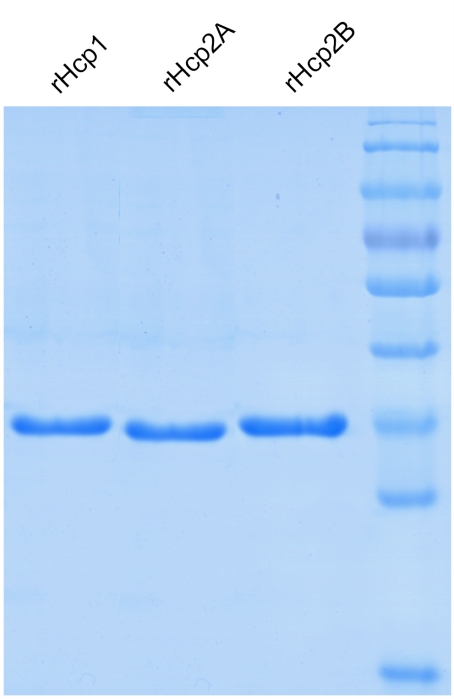
**

**Figure S2 Purification of** **recombinant Hcp (rHcp) proteins expressed in *E. coli* BL21 (DE3).** The whole ORFs of *hcps* were cloned into pET28a(+), and then induced the expression of about 29-kDa fusion protein Hcps. The expressed proteins were then purified two times using Ni-trap TM columns.
