# Supplementary material for: Three Hcp homologs with divergent extended loop regions exhibit different functions in avian pathogenic Escherichia coli
Source: Emerg Microbes Infect. 2018 Mar 29;7:49. doi: 10.1038/s41426-018-0042-0 (PMC5874247; doi:10.1038/s41426-018-0042-0)
Supplement: Supplementary file 3 — Supplementary Figure S3 [file 41426_2018_42_MOESM3_ESM.docx]

**
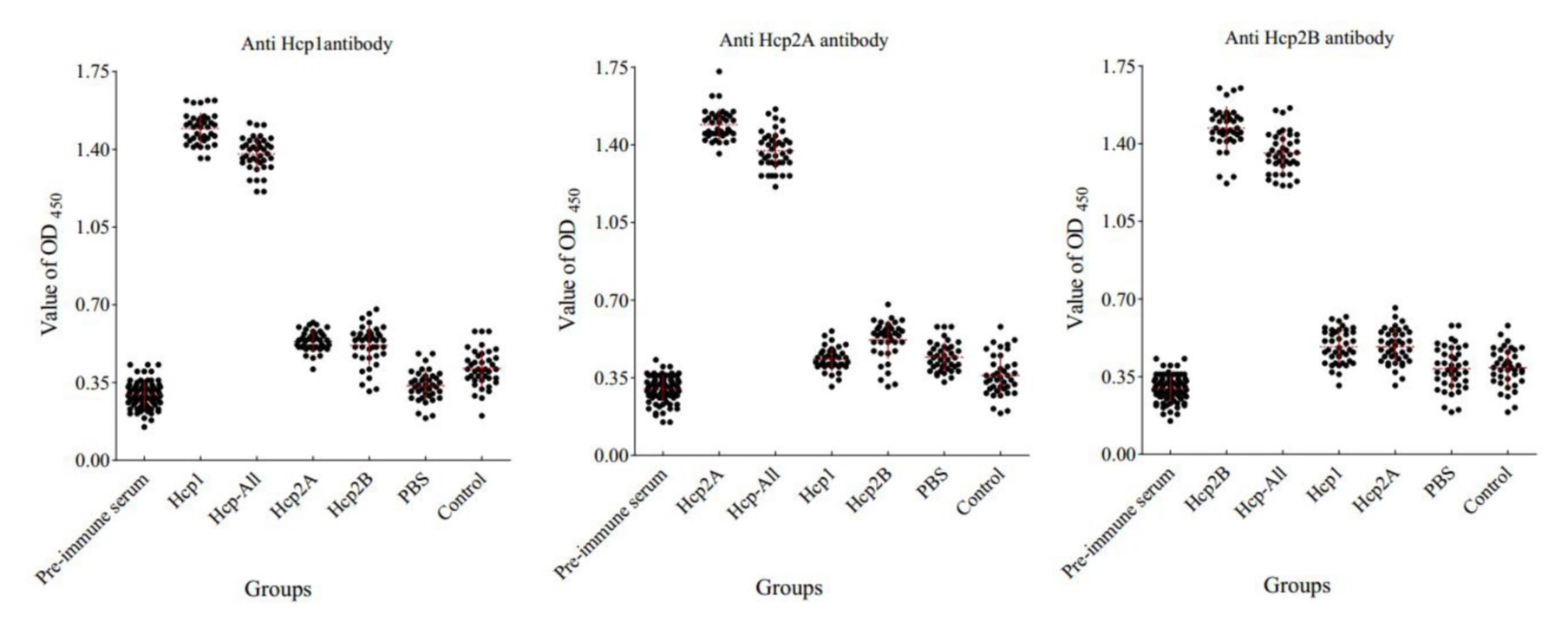
**

**Figure S3 IgG antibody titers of recombinant Hcps in immunized ducks.** Ducks were immunized with corresponding antigens emulsified in ISA 206 VG (100 µg/duck) at the primary and booster immunization (after 14 days) by back subcutaneous injection. The IgG antibody titers were determined by ELISA using a goat anti-duck IgG–HRP conjugate. Compared to the pre-immune serum, all groups immunized with recombinant proteins induced significantly higher IgG responses. ** *P* < 0.01, * *P* < 0.05.
