# Supplementary material for: Three Hcp homologs with divergent extended loop regions exhibit different functions in avian pathogenic Escherichia coli
Source: Emerg Microbes Infect. 2018 Mar 29;7:49. doi: 10.1038/s41426-018-0042-0 (PMC5874247; doi:10.1038/s41426-018-0042-0)
Supplement: Supplementary file 4 — Supplementary Figure S4 [file 41426_2018_42_MOESM4_ESM.docx]

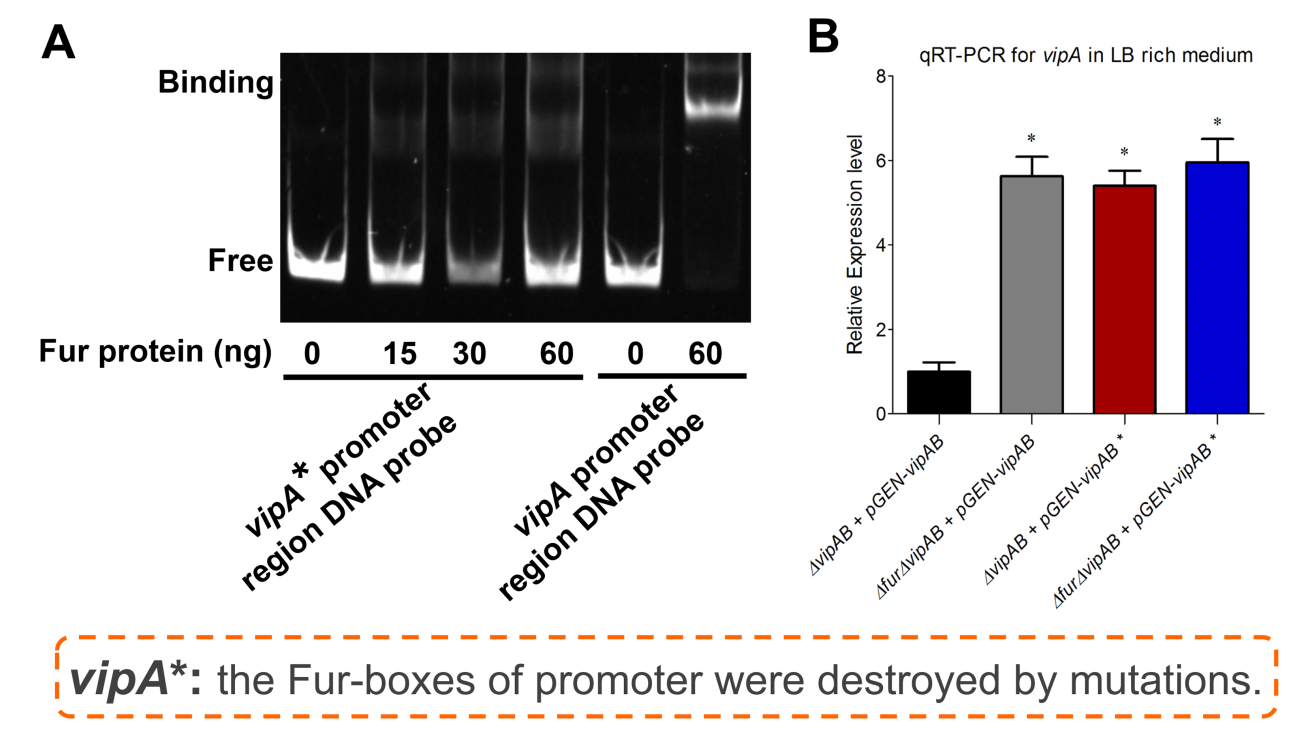


**Figure S4 The destruction of Fur-boxes significantly upregulated *vipA* gene expression in LB-rich medium.** (A) EMSA assays demonstrated that the *vipA** probes destroyed Fur-boxes could not be shifted by Fur fusion protein. The fragments containing the native promoter and *vipA* ORF were cloned into pGEN-MCS, and then reintroduced into corresponding mutant strains. The asterisk indicated that the Fur-boxes of native promoter were destroyed by several point mutations. The sequence containing point mutations was synthesized by Shanghai Sunny Biotechnology Co., Ltd. (B) The genes expression was analyzed by qRT-PCR (**p* < 0.05). The data were normalized to the housekeeping gene *tus* transcript. The relative expression levels represented the mean ± SD for three independently isolated RNA samples.
