# Supplementary material for: Three Hcp homologs with divergent extended loop regions exhibit different functions in avian pathogenic Escherichia coli
Source: Emerg Microbes Infect. 2018 Mar 29;7:49. doi: 10.1038/s41426-018-0042-0 (PMC5874247; doi:10.1038/s41426-018-0042-0)
Supplement: Supplementary file 5 — Supplementary Figure S5 [file 41426_2018_42_MOESM5_ESM.docx]

**
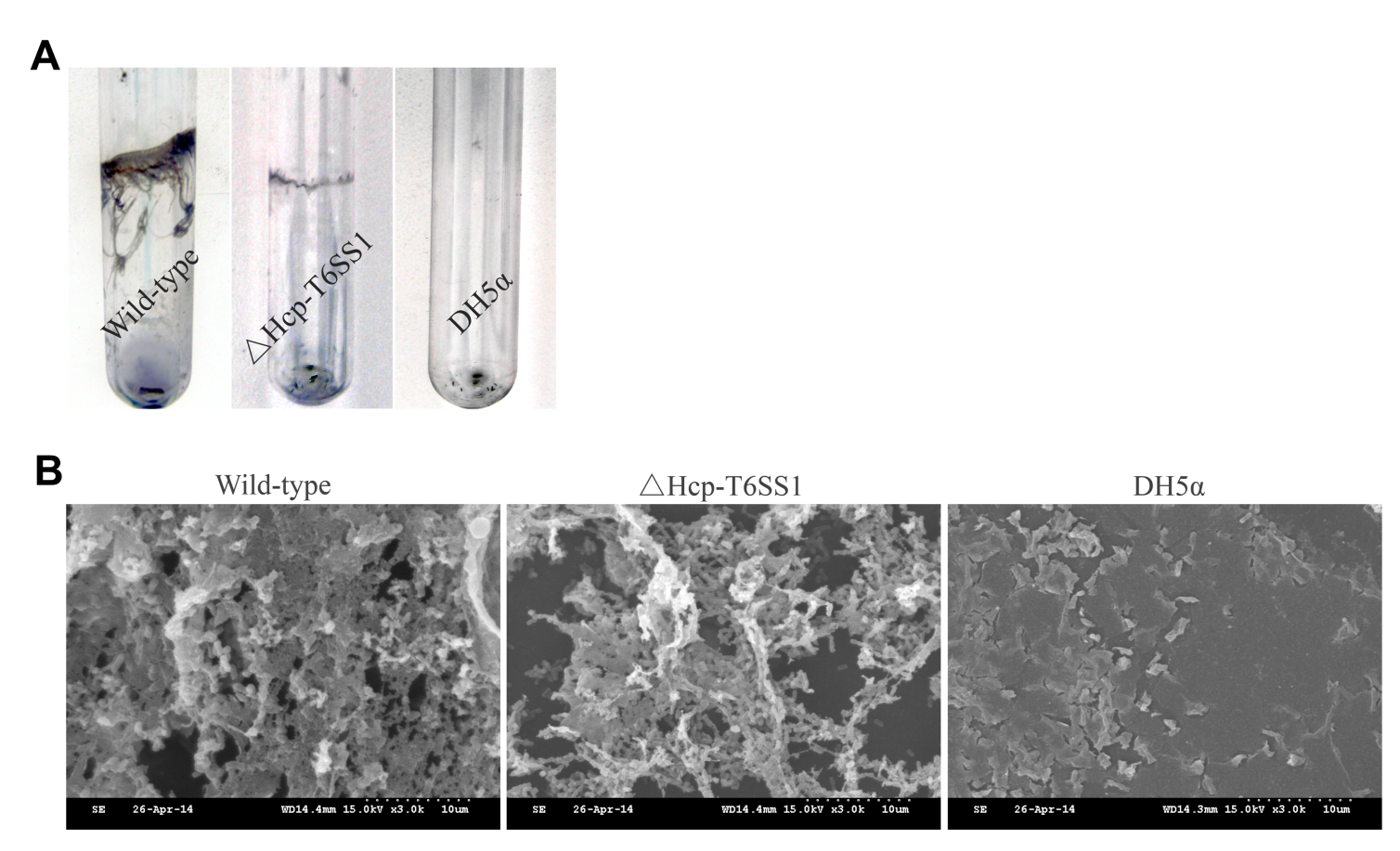
**

**Figure S5 Hcp1 was involved in APEC biofilm formation in host serum.** (A) Biofilms were visualized in glass tubes by crystal violet staining. (B) The images of the biofilm formation with scanning electron microscopy (SEM), at a magnification of ×3,000.
