# Supplementary figures and images for: Three Hcp homologs with divergent extended loop regions exhibit different functions in avian pathogenic Escherichia coli
Source: Emerg Microbes Infect. 2018 Mar 29;7:49. doi: 10.1038/s41426-018-0042-0 (PMC5874247; doi:10.1038/s41426-018-0042-0)

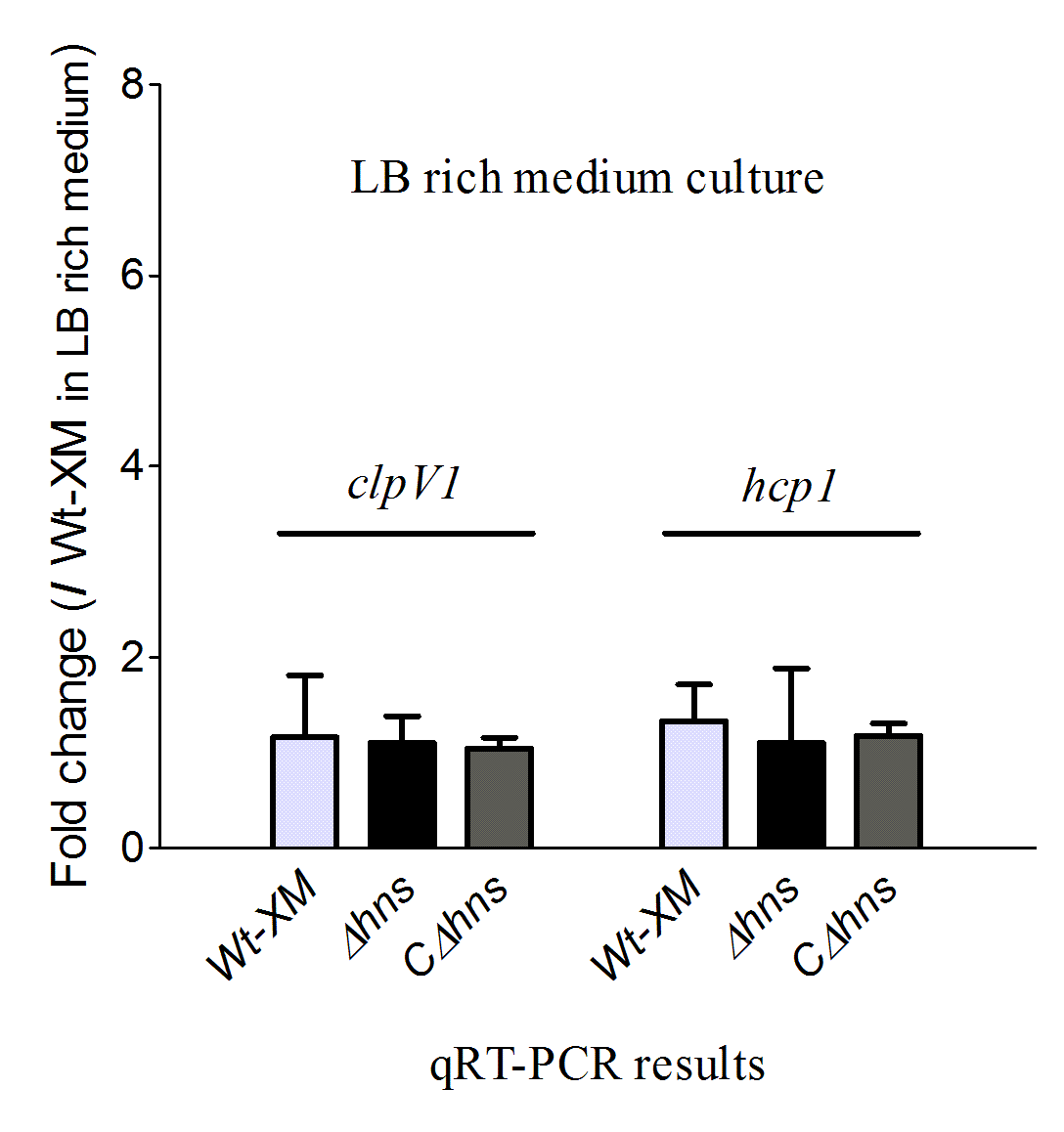


**Figure S6 The inactivation of H-NS did not active the transcription of the *hcp1-clpV1-vgrG1* cluster.**

Supplement: Supplementary file 6 — Supplementary Figure S6 [file 41426_2018_42_MOESM6_ESM.docx]
