# Supplementary material for: Three Hcp homologs with divergent extended loop regions exhibit different functions in avian pathogenic Escherichia coli
Source: Emerg Microbes Infect. 2018 Mar 29;7:49. doi: 10.1038/s41426-018-0042-0 (PMC5874247; doi:10.1038/s41426-018-0042-0)
Supplement: Supplementary file 7 — Supplementary Figure S7 [file 41426_2018_42_MOESM7_ESM.docx]

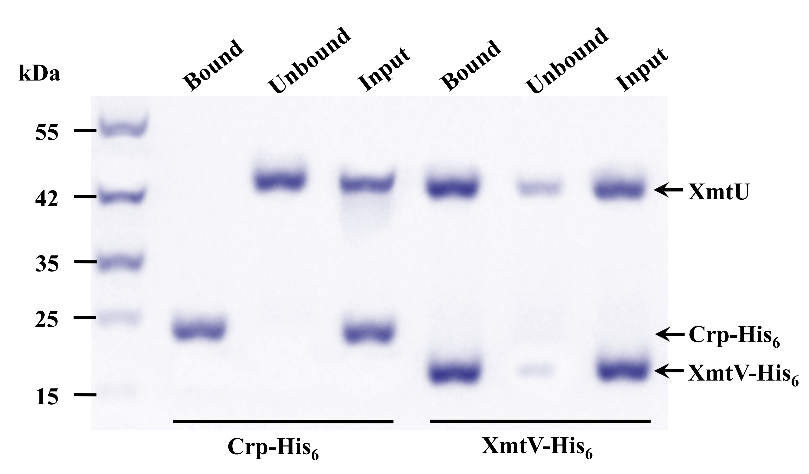


**Figure S7 Analysis of XmtU/XmtV binding by a protein pull-down assay as described previously.**[**^1^**](#_ENREF_1)**^,^** [**^2^**](#_ENREF_2)**{Ma, 2017 #46}** Purified XmtV-His6 and XmtU proteins were mixed at equimolar ratios and then purified by Ni^2+^-affinity chromatography. The Crp-His6 protein was used as a control in this experiment. All fractions were analyzed by SDS-PAGE and Coomassie blue staining.

1. Ma J, Pan Z, Huang J, Sun M, Lu C, Yao H. The Hcp proteins fused with diverse extended-toxin domains represent a novel pattern of antibacterial effectors in type VI secretion systems. *Virulence* 2017 Oct 3; **8**(7)**:** 1189-1202.

2. Ma J, Sun M, Dong W, Pan Z, Lu C, Yao H. PAAR-Rhs proteins harbor various C-terminal toxins to diversify the antibacterial pathways of type VI secretion systems. *Environmental microbiology* 2017 Jan; **19**(1)**:** 345-360.
