# Supplementary material for: Three Hcp homologs with divergent extended loop regions exhibit different functions in avian pathogenic Escherichia coli
Source: Emerg Microbes Infect. 2018 Mar 29;7:49. doi: 10.1038/s41426-018-0042-0 (PMC5874247; doi:10.1038/s41426-018-0042-0)
Supplement: Supplementary file 8 — Supplementary Figure S8 [file 41426_2018_42_MOESM8_ESM.docx]

**
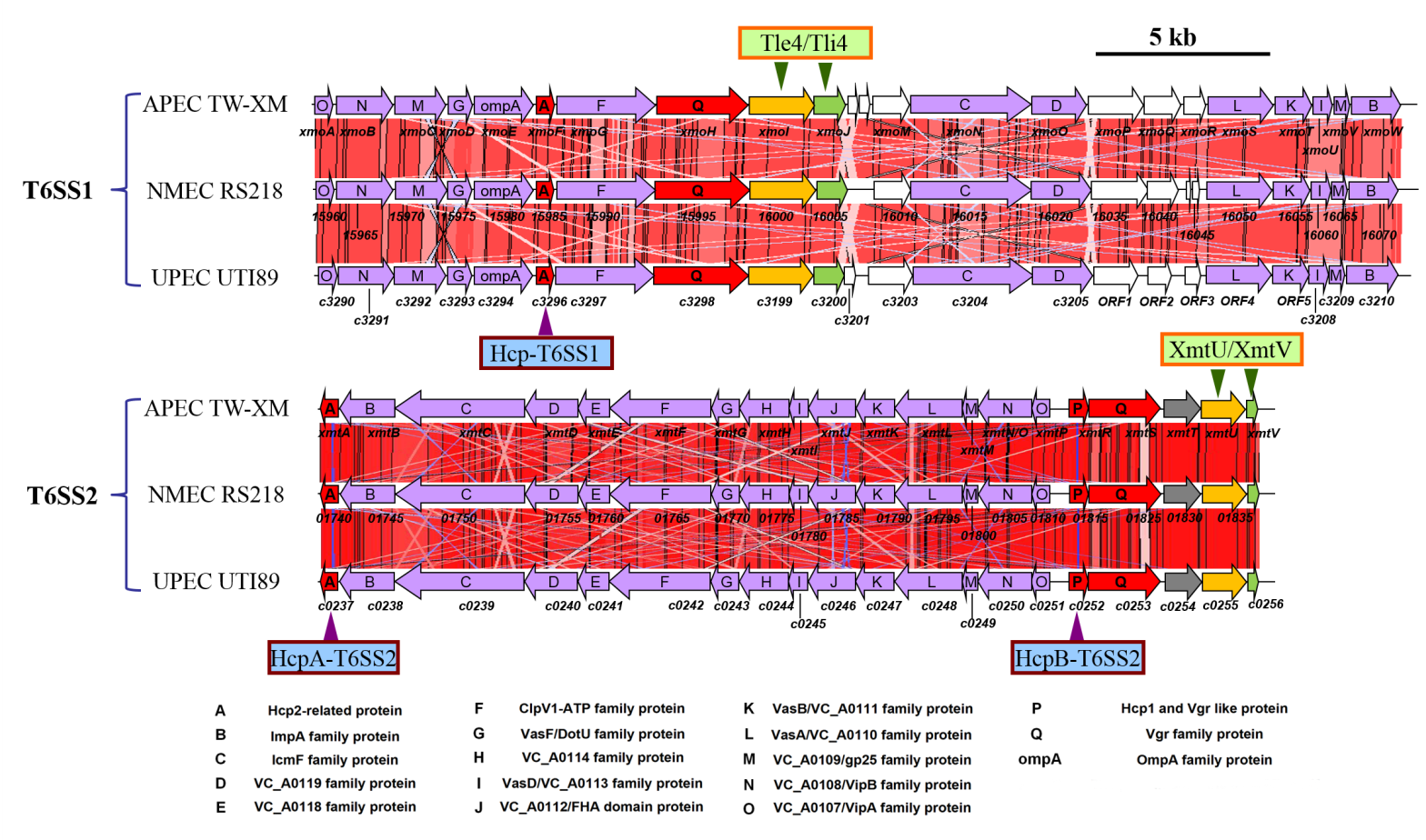
**

**Figure S8 Comparative genome alignments of T6SS1 and T6SS2 loci from APEC TW-XM, NMEC RS218, and UPEC UTI89.** The nucleotide sequences of T6SS loci were downloaded from NCBI, and the GenBank accession numbers were KF678349 (T6SS1 locus of TW-XM), KF678350 (T6SS2 locus of TW-XM), NC_007946 (UTI89) and CP007149 (RS218). Visual representation of the alignments by using nucleotide similarities (tblastx) of the T6SS loci was carried out with the Artemis Comparison Tool (ACT).
