# Supplementary material for: Three Hcp homologs with divergent extended loop regions exhibit different functions in avian pathogenic Escherichia coli
Source: Emerg Microbes Infect. 2018 Mar 29;7:49. doi: 10.1038/s41426-018-0042-0 (PMC5874247; doi:10.1038/s41426-018-0042-0)
Supplement: Supplementary file 9 — Supplementary Table S1 [file 41426_2018_42_MOESM9_ESM.docx]

**Table S1 Summary of bacterial strains and plasmids.**

| Strain or plasmid | Characteristic(s) | Source |
| --- | --- | --- |
| **Strains** | | |
| TW-XM | O2:K1; isolated from a duck with neurological symptoms; *lacZ*^+^, T6SS1^+^, T6SS2^+^ | [^1^](#_ENREF_1) |
| Δ*tle4* | Deletion mutant of *tle4* from T6SS1 with TW-XM background | [^1^](#_ENREF_1) |
| Δ*tle4*Δ*tli4* | Deletion mutant of *tle4-tli4* from T6SS1 with TW-XM background *lacZ*^−^ | [^1^](#_ENREF_1) |
| Δ*vgrG2* | Deletion mutant of *vgrG2* from T6SS2 with TW-XM background | [^1^](#_ENREF_1) |
| Δ*xmtU* | Deletion mutant of *xmtU* from T6SS2 with TW-XM background | This study |
| CΔ*xmtU* | The Δ*xmtU* strain contains the pGEN-*xmtU* | This study |
| Δ*xmtU*Δ*xmtV* | Deletion mutant of *xmtU-V* from T6SS2 with TW-XM background *lacZ*^−^ | This study |
| Δ*clpV1*&*clpV2* | Deletion mutant of two *clpVs* from T6SS1 and T6SS2 with TW-XM background | This study |
| Δ*hcp1* | Deletion mutant of *hcp1* from T6SS1 with TW-XM background | This study |
| Δ*hcp2A* | Deletion mutant of *hcp2A* from T6SS2 with TW-XM background | This study |
| Δ*hcp2B* | Deletion mutant of *hcp2B* from T6SS2 with TW-XM background | This study |
| CΔ*hcp1* | TW-XM Δ*hcp1* with the vector pGEN-*hcp1* | This study |
| Δ*fur* | Deletion mutant of *fur* with TW-XM background | This study |
| CΔ*fur* | TW-XM Δ*fur* with the vector pGEN-*fur* | This study |
| Δ*hns* | Deletion mutant of *hns* with TW-XM background | This study |
| CΔ*hns* | TW-XM Δ*hns* with the vector pGEN-*hns* | This study |
| Δ*vipAB* | Deletion mutant of *vipAB* with TW-XM background | This study |
| Δ*fur*Δ*vipAB* | Deletion mutant of *vipAB* with TW-XM Δ*fur* background | This study |
| *E. coli* DH5α | Cloning host for maintaining the recombinant plasmids | Invitrogen |
| *E. coli* BL21 | Host for expressing the recombinant proteins | Invitrogen |
| **Plasmids** |  |  |
| pET21a(+) | Expression vector, Kan^R^ | Novagen |
| pGEN MCS | *E. coli* shuttle vector; Amp^R^ | Laboratory stock |
| pET21a-*fur* | pET21a carrying *fur* coding region | This study |
| pGEN-*hcp1* | pGEN MCS carrying *hcp1* under the control of Pcm promoter | This study |
| pGEN-*hcp1^DelVs2^* | pGEN MCS carrying *hcp1* without Vs2 region under the control of Pcm promoter | This study |
| pGEN-*hcp2A* | pGEN MCS carrying *hcp2A* under the control of Pcm promoter | This study |
| pGEN-*hcp2A^DelVs1^* | pGEN MCS carrying *hcp2A* without Vs1 region under the control of Pcm promoter | This study |
| pGEN-*hcp2A* | pGEN MCS carrying *hcp2B* under the control of Pcm promoter | This study |
| pGEN-*hcp2B^DelVs1^* | pGEN MCS carrying *hcp2B* without Vs1 region under the control of Pcm promoter | This study |
| pGEN-*hcp2B^DelVs2^* | pGEN MCS carrying *hcp2B* without Vs2 region under the control of Pcm promoter | This study |
| pGEN-*xmtU* | pGEN MCS carrying *xmtU* gene under the control of Pcm promoter | This study |
| pGEN-*fur* | pGEN MCS carrying *fur* gene under the control of Pcm promoter | This study |
| pGEN-*hns* | pGEN MCS carrying *hns* gene under the control of Pcm promoter | This study |
| pKD46 | Red recombinase expression plasmid | [^2^](#_ENREF_2) |
| PKD4 | pANTS derivative containing FRT-flanked kanamycin resistance | [^2^](#_ENREF_2) |
| pCP20 | TS replication and thermal induction of FLP synthesis | [^2^](#_ENREF_2) |

Vs1: Variant sequence 1, locate in loop 1, 2, from Ala19 to Ser27 according to the Hcp3-Pa amino acid sequence in Fig. 6;

Vs2: Variant sequence 2, locate in loop 2, 3, from Ser56 to His66 according to the Hcp3-Pa amino acid sequence in Fig. 6.

1. Ma J, Bao Y, Sun M, Dong W, Pan Z, Zhang W*, et al.* Two functional type VI secretion systems in avian pathogenic Escherichia coli are involved in different pathogenic pathways. *Infect Immun* 2014 Sep; **82**(9)**:** 3867-3879.

2. Datsenko KA, Wanner BL. One-step inactivation of chromosomal genes in Escherichia coli K-12 using PCR products. *Proceedings of the National Academy of Sciences of the United States of America* 2000 Jun 06; **97**(12)**:** 6640-6645.
