# Supplementary material for: Three Hcp homologs with divergent extended loop regions exhibit different functions in avian pathogenic Escherichia coli
Source: Emerg Microbes Infect. 2018 Mar 29;7:49. doi: 10.1038/s41426-018-0042-0 (PMC5874247; doi:10.1038/s41426-018-0042-0)
Supplement: Supplementary file 10 — Supplementary Table S2 [file 41426_2018_42_MOESM10_ESM.docx]

**Table S2. Primers used for PCR amplification, gene deletion and qRT-PCR.**

| Primer | Sequence(5'-3') |
| --- | --- |
| ***General PCR for cloning*** | |
| Fur-F | GGCGGATCCACTGATAACAATACCGCCCTA |
| Fur-R | GTGTCTAGATTTGCCTTCGTGCGCGTGCTCA |
| H-NS-F | GGCGGATCCATGAGCGAAGCACTTAAAATTCTG |
| H-NS-R | GTGTCTAGATTATTGCTTGATCAGGAAATC |
| Hcp1-F | GGCGAATTCATTCCTGCTTATCTCTGGCTG |
| Hcp1-R | CCCAAGCTTTTAAGGACGCTCATTCCACGAGTC |
| Hcp2A-F | GGCGAATTCATGGCAAATCCCGTCTATCTGA |
| Hcp2A-R | CCCAAGCTTTTAGAAAATCCGTTCACTCCAA |
| Hcp2B-F | GGCGAATTCATGCCAACCCCATGTTACAT |
| Hcp2B-R | CCCAAGCTTTTATGCTTCCAGCGGTGCAC |
| pGEN-Hcp1-F | ATCGGATCCGCAATCAGGTCAAAATTCCG |
| pGEN-Hcp1-R | CTCGTCGACGTTCAGGCACGCTACCGCCCAT |
| pGEN-Hcp2A-F | ATCGGATCCATTGTCCTCTCAACATATAAGC |
| pGEN-Hcp2A-R | CTCGTCGACCGGTTACTGAATCAAGAAACAC |
| pGEN-Hcp2B-F | ATCGGATCCATCGTGTTCTGAAATTGACT |
| pGEN-Hcp2B-R | CTCGTCGACTAATGACCTCCCTGATTCATTC |
| ***For Deletion^a^*** | |
| Del-Hcp1-F | CGCCGGAAGAAGGCGGTGCTTCAATCACACTAACAAGGAGAGTAATTCTCgtgtaggctggagctgcttc |
| Del-Hcp1-R | GGACAAAGCACACAACAAAAAGGCAGACAGTTTCCTGTCTGCCGCAGTTAatgggaattagccatggtcc |
| Del-Hcp2A-F | AACAGAACCGAACCTGTCCATCCAACTCATTGAGATTAAGGAGAATAACAgtgtaggctggagctgcttc |
| Del-Hcp2A-R | TTGCCTTTTTAAAATATAACAATAATGCAGATGAAAGACTCCCTGGTAACatgggaattagccatggtcc |
| Del-Hcp2B-F | GACGGGTTGTTCGTAAAACAGCAGTTGATAATTTCACAAGGAGTTCATAAgtgtaggctggagctgcttc |
| Del-Hcp2B-R | ATCTGAGTCAACAACATCCTGCCTCCGTGCAGGATGTTGTTTTTGTACGTatgggaattagccatggtcc |
| Del-Fur-F | TCTCGTTCAGGCTGGCTTATTTGCCTTCGTGCGCGTGCTCATCTTCGCGGgtgtaggctggagctgcttc |
| Del-Fur-R | TGAAGTGAACCGCTTAGTAACAGGACAGATTCCGCATGACTGATAACAATatgggaattagccatggtcc |
| Del-H-NS-F | AAACCACCCCAATATAAGTTTGAGATTACTACAATGAGCGAAGCACTTAAgtgtaggctggagctgcttc |
| Del-H-NS-R | CCCTCGACGATTTCCTGATCAAGCAATAATCTTTTGTAGATTGCACATGCatgggaattagccatggtcc |
| Del-ClpV1-F | GTTTCCAGAATGAAGTTCCCACCGCTCGTGTAAATATCAAGCTTGATCTGgtgtaggctggagctgcttc |
| Del-ClpV1-R | CGTCTTCCCCATACTCTTCCGCATCCTTCATACTCCTGCCGGTCAGAAATatgggaattagccatggtcc |
| Del-ClpV2-F | GGATGGACAGGTTCGGTTCTGTTATCCGTTGCCATATCCTGTCCGGTATTgtgtaggctggagctgcttc |
| Del-ClpV2-R | TTGCCTTTATGTCCGGTAGCCTGCGCCAGAAACTTGAGGACGAATTCCCCatgggaattagccatggtcc |
| ***Primers for reverse transcription qRT-PCR*** | |
| tus-qPCR-F | TCGTAGACCGACTCAACACTA |
| tus-qPCR-R | TTACCTCCGGCAAAGAGAAC |
| hcp1-qPCR-F | CGGTCAGGAAGTGGAGTATTT |
| hcp1-qPCR-R | GCTTCTCTTTACCCGGATCTT |
| vgrG1-qPCR-F | CAGCGTGTTGAAGAAGTTTACC |
| vgrG1-qPCR-R | CTGCGCCTGAATCTCTACTT |
| vgrG2-qPCR-F | CGAAGACGCAGATGACGATAC |
| vgrG2-qPCR-R | GCGTGGATATAGACCTGTTCAC |
| hcp2A-qPCR-F | ACGAAACCGGTGGACAAA |
| hcp2A-qPCR-R | GGTTGGTGCGGTAGAATACA |
| icmF2-qPCR-F | CGCGCTGGAGCAGATTAT |
| icmF2-qPCR-R | CCTCGCCTTATCATCCAGTTT |
| clpV2-qPCR-F | GAGACGCTCGCTACCATTATT |
| clpV2-qPCR-R | TGATTTCGTCCGTCACTTCC |
| vipA2-qPCR-F | TAACACGCCGTTGGATGAG |
| vipA2-qPCR-R | GTTCAGCCGGAACAACAAAC |
| hcp2B-qPCR-F | GTGAAATGCTGCCGAAAGTG |
| hcp2B-qPCR-R | ACAATCGTCGCGTCAGTAAG |
| ***Primers for EMSA*** | |
| T6SS1-F | TGCAAATTTGTGAAGCTTATC |
| T6SS1-R | ATCCTTTTCCTCATCATCCAC |
| FepA-F | GCCACCAGAAAGTGACCTCAAA |
| FepA-R | TGATCCTGAAAGACACGCAGTG |
| Negative-F | CTTGCCGTAGGCGATTACAGT |
| Negative-R | GGATTTCAGGTCACGTAGTAA |

Underlined showed restriction cutting sites;

The genes fragments of *hcp1^DelVs2^*, *hcp2A^DelVs1^*, *hcp2B^DelVs1^*, *hcp2B^DelVs2^* were synthesized by Shanghai Personal Biotechnol. Co., Ltd.
