# Supplementary material for: Three Hcp homologs with divergent extended loop regions exhibit different functions in avian pathogenic Escherichia coli
Source: Emerg Microbes Infect. 2018 Mar 29;7:49. doi: 10.1038/s41426-018-0042-0 (PMC5874247; doi:10.1038/s41426-018-0042-0)
Supplement: Supplementary file 11 — Supplementary data [file 41426_2018_42_MOESM11_ESM.docx]

**Supplementary data**
**Figure S1** Evolutionary relationships among 136 known and putative Hcps from gram-negative bacteria.

**Figure S2** Purification of recombinant Hcp proteins expressed in *E. coli* BL21 (DE3).

**Figure S3** IgG antibody titers of recombinant Hcps in immunized ducks.

**Figure S4** The destruction of Fur-boxes significantly up-regulated *vipA* gene expression in LB-rich medium.

**Figure S5** Hcp1 was involved in APEC biofilm formation in host serum.

**Figure S6** The inactivation of H-NS did not activate transcription of the *hcp1*-*clpV1-vgrG1* cluster.

**Figure S7** Analysis of XmtU/XmtV binding by a protein pull-down assay as described previously.

**Figure S8** Comparative genome alignments of T6SS1 and T6SS2 loci from APEC TW-XM, NMEC RS218, and UPEC UTI89.

**Table S1** Summary of bacterial strains and plasmids.

**Table S2.** Primers used for PCR amplification, gene deletion, and reverse transcription qRT-PCR.
